# Focusing on mixed narrow band stimuli: Implications for mechanisms of accommodation and displays

**DOI:** 10.1167/jov.24.9.14

**Published:** 2024-09-20

**Authors:** Abigail P. Finch, Maydel Fernandez-Alonso, Andrew K. Kirby, Jenny C. A. Read, Gordon D. Love

**Affiliations:** 1 Department of Physics, Durham University, Durham, UK; 2 Biosciences Institute, Newcastle University, Newcastle, UK, Present address: Max Planck Institute for Biological Cybernetics, Germany; 3 Department of Physics, Durham University, Durham, UK; 4 Biosciences Institute, Newcastle University, Newcastle, UK; 5 School of Computer Science, University of Leeds, Leeds, UK

**Keywords:** accommodation, displays and vision, color vision

## Abstract

The eye has considerable chromatic aberration, meaning that the accommodative demand varies with wavelength. Given this, how does the eye accommodate to light of differing spectral content? Previous work is not conclusive but, in general, the eye focuses in the center of the visible spectrum for broadband light, and it focuses at a distance appropriate for individual wavelengths for narrowband light. For stimuli containing two colors, there are also mixed reports. This is the second of a series of two papers where we investigate accommodation in relation to chromatic aberration Fernandez-Alonso, Finch, Love, and Read (2024). In this paper, for the first time, we measure how the eye accommodates to images containing two narrowband wavelengths, with varying relative luminance under monocular conditions. We find that the eye tends to accommodate between the two extremes, weighted by the relative luminance. At first sight, this seems reasonable, but we show that image quality would be maximized if the eye instead accommodated on the more luminous wavelength. Next we explore several hypotheses as to what signal the eye might be using to drive accommodation and compare these with the experimental data. We show that the data is best explained if the eye seeks to maximize contrast at low spatial frequencies. We consider the implication of these results for both the mechanism behind accommodation, and for modern displays containing narrowband illuminants.

## Introduction

Accommodation is the process by which the crystalline lens within the eye changes shape and, in doing so, changes the distance at which objects appear in focus. Due to longitudinal chromatic aberration (LCA), the optical power of the lens changes as a function of wavelength, so only one wavelength of light can be in focus on the retina at any one time. Therefore, it is possible that the eye could accommodate differently for different chromatic stimuli. For example, for a stimulus with a spectrum biased toward longer wavelengths, it seems reasonable to assume that the eye might accommodate to bring the longer wavelengths into focus and not the shorter ones and vice versa for a stimulus mostly composed of shorter wavelengths. It is also conceivable that, depending on the cues the eye uses to accommodate, there may be certain spectra for which the eye does not accommodate optimally.

In the modern world we are increasingly exposed to unnatural illuminant spectra, for example, in some LED lights. These are often made up of a series of narrowband peaks, rather than having a smooth, broadband intensity distribution as a function of wavelength. One effect of these modern types of illuminant is that they may alter the appearance of object colors and impair our color constancy. This is often considered when selecting illuminants and attempts have been made to quantify this effect using a color rendering index. In contrast, we know very little about the effect that certain modern illuminants might have on the optimal accommodation response, people's actual accommodation responses, and ultimately on retinal image blur, and we have no equivalent to the color rendering index for quantifying and minimizing these.

### Previous research

#### Accommodation to different spectra

In narrowband or monochromatic light it has been found that some observers can adjust their accommodation to compensate for the LCA of the eye (Charman & Tucker, 1978; Fernandez-Alonso, Finch, Love, & Read, 2024). This means that the static accommodation response will differ depending on the wavelength for stimuli presented at the same distance.

There are mixed findings in the literature regarding accommodation under broadband white illumination. Ivanoff (1949) found that the focusing wavelength in white light shifts with accommodation from almost 700 nm when the stimulus is presented at infinity to approximately 500 nm when the stimulus is presented at 2.5 D. However, it may be that in this case what was in fact being measured is the apparent lead and lag in the accommodation response function.

Both Charman and Tucker (1978) and Lovasik and Kergoat (1988) found that the static accommodation position for white light was similar to that for green light. However, in a different type of study, DeHoog and Schwiegerling (2007) allowed subjects to adjust the focus of a white light source and a series of monochromatic sources and found that the selected best focus for white light was generally equivalent to that for monochromatic light between 590 and 610 nm, which is more in the orange or red part of the spectrum. Coe, Bradley, and Thibos (2014) measured the wavelength subjects focus at when observing white light and found it equals monochromatic refractive error for 569 nm for a 3-mm pupil and 575 nm for an 8 mm pupil. It may be that these differences in findings are due to the different white light spectra used and different viewing distances.

In a parallel paper (Fernandez-Alonso et al., 2024) to this, we also measured the static accommodation responses for white light compared to that for narrowband light at various wavelengths. We found that the static accommodation responses for white light generally varied somewhere between that for green light (527 nm) and orange light (588 nm). This wavelength range aligns roughly with the peak of the luminous efficiency function. Therefore, it seems reasonable to assume that in white light we accommodate around the wavelengths that we are most sensitive to in the spectrum.

In terms of stimuli containing two colors, von Bahr (1946) measured visual acuity with two narrowband lights and found that it is similar with two sources to just one. Lovasik and Kergoat (1988) ran an experiment in which they investigated accommodation responses to blue letters on a red background, blue letters on a green background, and red letters on a green background. They found no clear difference in the accommodative response for these three stimuli. Responses for all three of these mixtures (even the blue letters on a green background) were similar to that for red letters on a black background, and sometimes even greater. Similarly Wolfe and Owens (1981) and Switkes, Bradley, and Schor (1990) found that accommodation wasn’t driven by a chromatic border. Charman (1989) ran an experiment in which they measured accommodative responses to a blue on a red background and a red on a blue background. They found that observers always accommodated to either the red light or the blue light and never in the middle of the two. From these experimental data we might expect that for spectra made up of two peaks at different wavelengths, observers will not focus in between the two wavelengths, but rather at around one wavelength or the other.

#### Accommodation to multiplane displays and bifocal contact lenses

There are parallels between our work and other work on both multiplane displays and bifocal contact lenses. Both involve presenting the visual system with different images of varying focus. A multiplane display (Akeley, Watt, Girshick, & Banks, 2004) is one that displays stimuli at different distances from the eye. It is possible to drive accommodation in between two of the planes by manipulating the intensity ratio between the two planes by a process known as depth-weighted filtering (Watt, Akeley, Girshick, & Banks, 2005). In this process, the intensity of the image in each stimulus plane is determined by the distance of the desired simulated image plane from the actual stimulus plane. So if the desired stimulus distance is closer to one plane than the other, then stimuli on that plane must be made brighter. MacKenzie and Watt (2010) found that for image separations up to 1.11 diopters (D), accommodation could be driven continuously and almost linearly using depth-weighted filtering. However, at larger image plane separations they found the accommodation response to be biased toward one of the two planes.

Similarly, bifocal contact lenses can provide mixed focus stimuli. Such a lens consists of two zones of differing optical power. The idea is that presybopic subjects will select the image with the best focus so that they can view both near and far objects. Experiments with younger subjects who can still accommodate have shown that they tend to accommodate in the middle (Altoaimi, Almutairi, Kollbaum, & Bradley, 2018).

Finally, our work has parallels with the duochrome test used by optometrists, in which case a subject is presented with a green and red image and asked which is in best focus.

These findings can be applied to accommodative responses to different spectra if we think of the different focal depths from different wavelengths, owing to LCA, as being analogous to the different stimulus planes from either a multiplane display or a bifocal contact lens For a spectrum with two peaks, as long as the difference in LCA between the two peaks is less than 1.1 D, we would expect the accommodation position to be somewhere in between the two peak wavelengths and to vary with the relative intensity of the two peaks in line with depth-weighted filtering.

### Present study

The aim of this study was to establish where people accommodate to spectra made up of a mixture of two narrowband components as a step to understanding how we might accommodate to modern spectra with multiple peaks. It may also offer an insight into the operation of the human accommodation system, for example, what it seeks to optimize.

We conducted an experiment measuring observers’ static accommodative responses to a stimulus illuminated from behind by various mixtures of narrowband LEDs. At any one time, the stimulus was only illuminated by one or two of the LEDs. Therefore, all of the spectra had either one or two peaks in intensity as a function of wavelength.

There were two possible hypotheses as to where people would accommodate for the mixed stimuli. The first was that people would accommodate to one of the two individual LEDs. This thinking is in line with the findings of Charman (1989), although there the different wavelengths were spatially separated rather than superimposed. The second hypothesis was that, as long as the dioptric separation between the two wavelengths owing to LCA was less than 1.1 D, as the intensity ratio between the two LEDs changed, there would be a roughly linear accommodation response between the two LEDs. This thinking is in line with the findings of MacKenzie and Watt (2010) on multiplane displays and Altoaimi et al. (2018) on multifocal contact lenses.

We then carried out a series of simulations to predict where accommodation might be driven for the stimuli used in the experiment. These simulations were run for a variety of different optimisation rules using a variety of potential optical cues to accommodation.

The aim of these simulations was to discover the best rule for predicting the measured accommodation responses found in the experiment. This rule could then be used to predict the accommodation responses to different chromatic stimuli and to gain an insight into the optical cues to accommodation used by the visual system.

## Methods

### Apparatus

**Figure 1.**
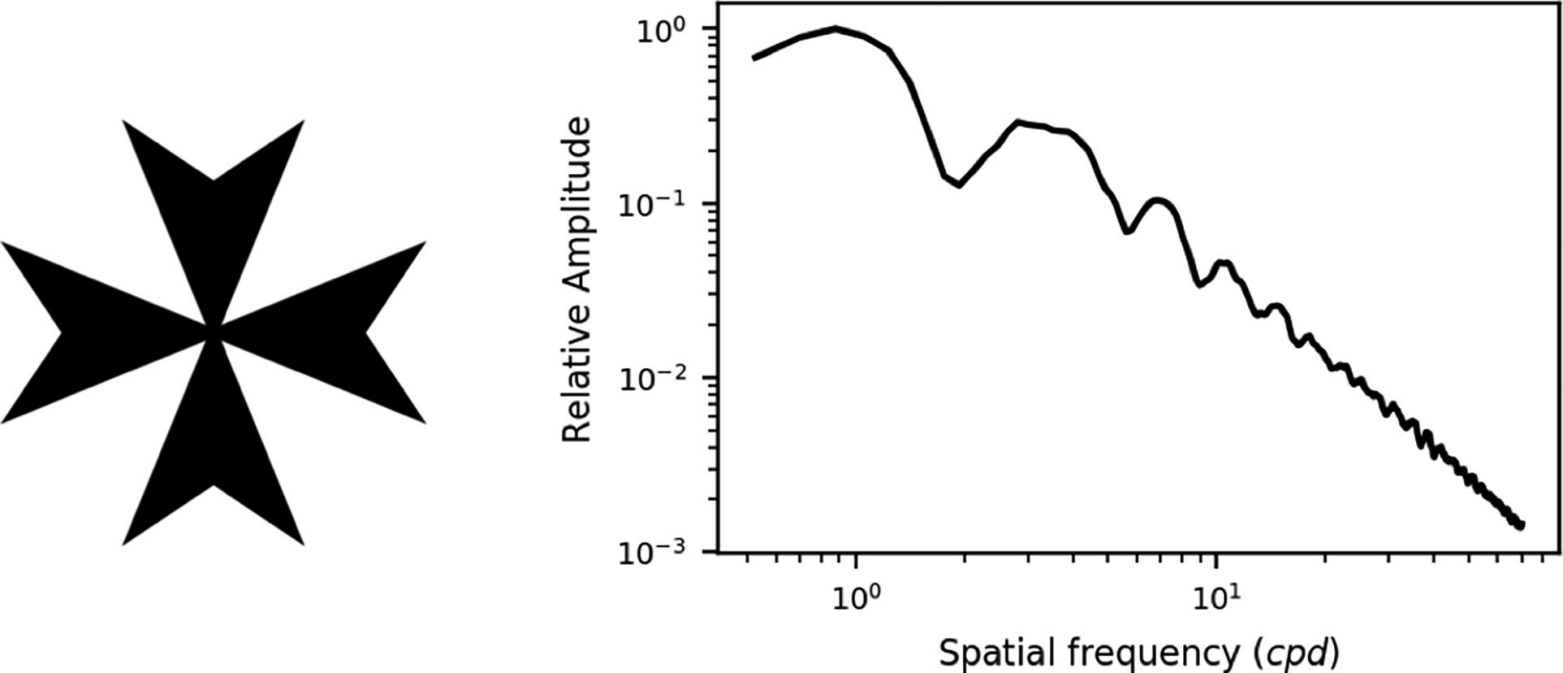
The fixation stimulus used in the experiment is shown on the left. The spatial frequency power spectrum is shown on the right. The stimulus size was 1.5° and the power spectrum was an azimuthal average around the stimulus.

**Figure 2.**
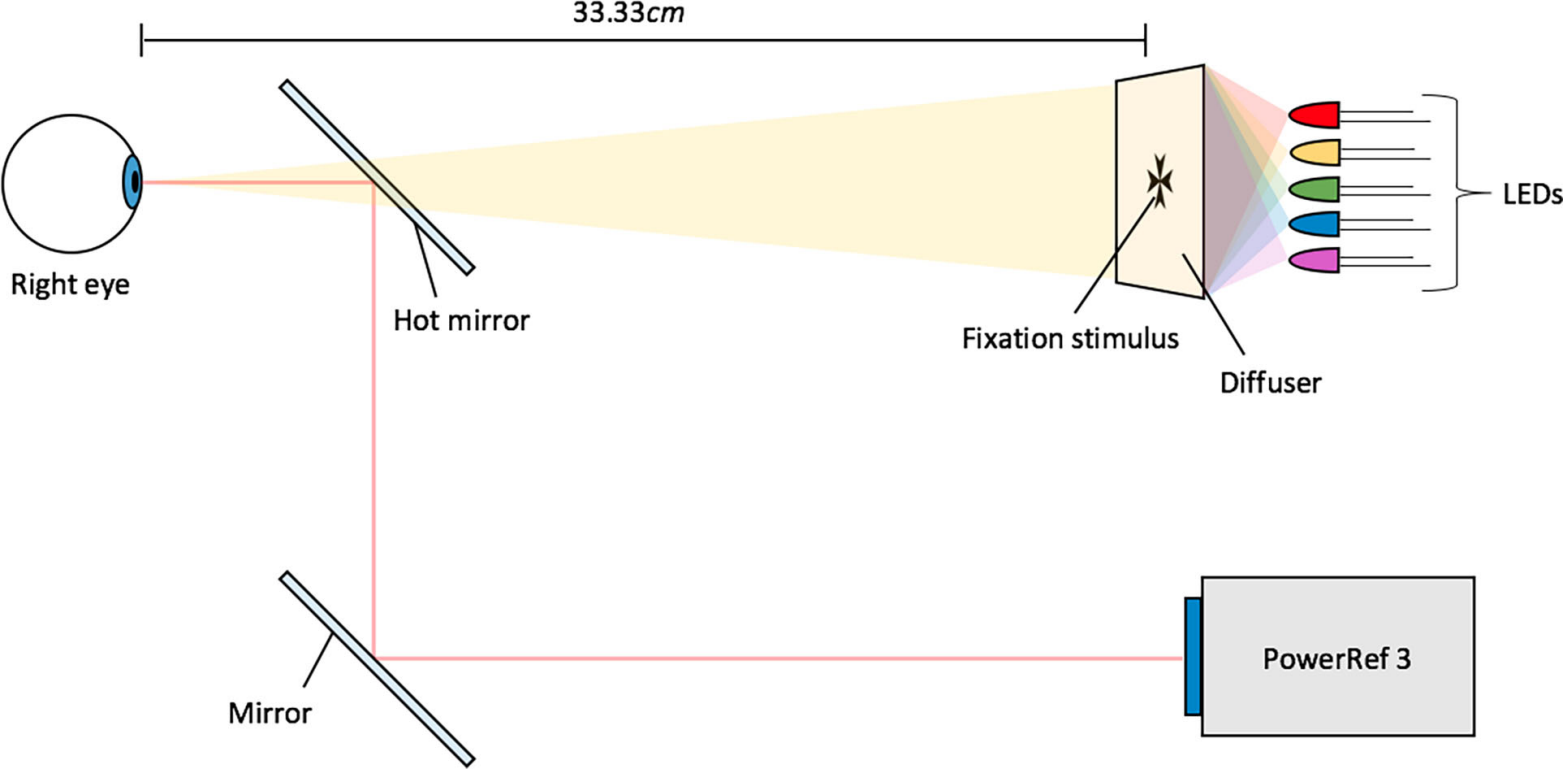
A diagram (not to scale) showing the setup of the apparatus. The periscope system, including a hot mirror, allows the participant to view the stimulus while the PowerRef 3 measures the refraction of the eye. The only difference in the setup for the calibration procedure was that the stimulus was at 100 cm and the fixation cross was larger to maintain the angular size.

The apparatus is described in detail in our other paper (Fernandez-Alonso et al., 2024). In brief, the stimulus was a black Maltese cross printed on transparency film and mounted on a diffuser. This was positioned 33 cm (3 D) away from the observer. At this distance the stimulus window subtended 2.6° of visual angle and the Maltese cross was 1.5° across. Figure 1 shows an image of the Maltese cross and its spatial frequency content, and a diagram of the set-up is shown in Figure 2. A Maltese cross was chosen as it has a broad range of spatial frequency components.

**Figure 3.**
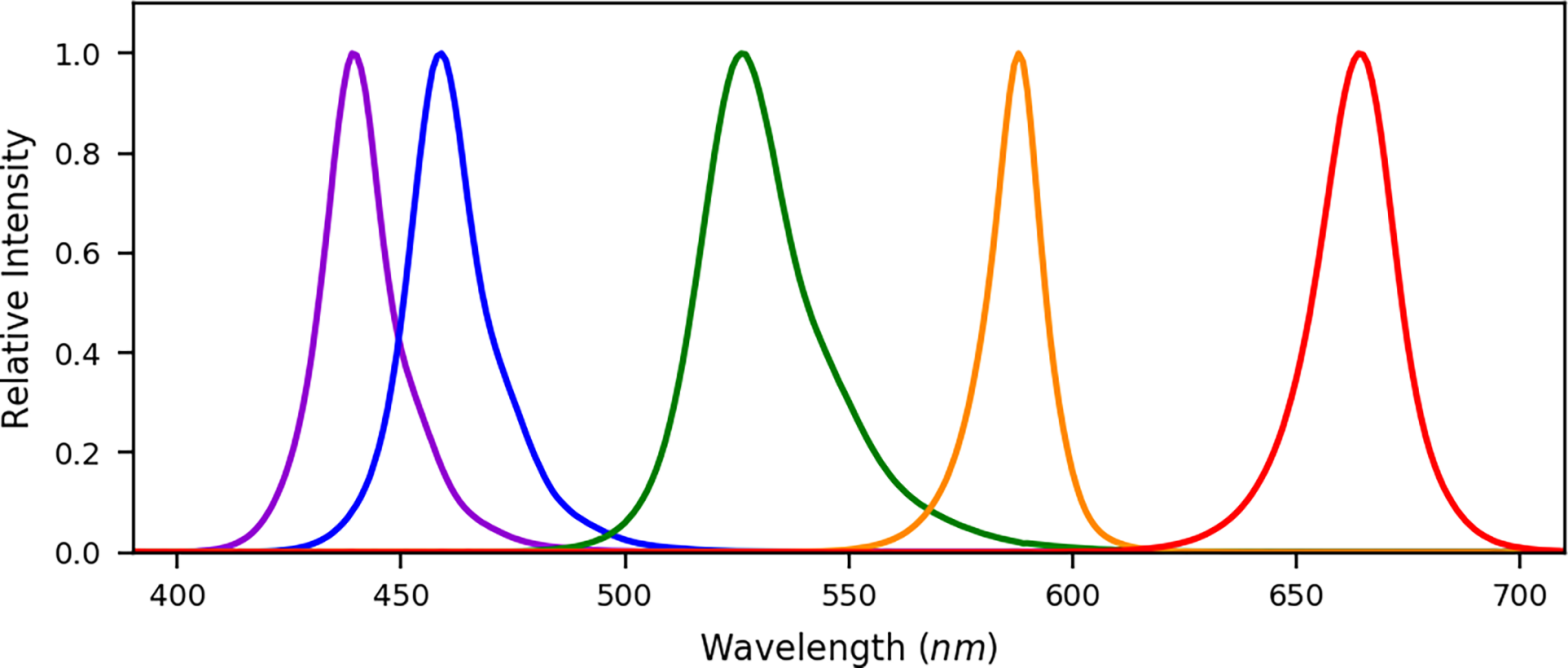
Radiance measures for each of the six LEDs. These have been normalised to have peak values of one. The colors shown for each are a guide for the eye with red (peak: 660 nm), orange (peak: 588 nm), green (peak: 527 nm), blue (peak: 461 nm), and violet (peak: 441 nm).

Behind the diffuser there were five narrowband LEDs whose spectra are plotted in Figure 3. From now on these LEDs will be referred to as red (peak: 660 nm), orange (peak: 588 nm), green (peak: 527 nm), blue (peak: 461 nm), and violet (peak: 441 nm), with spectral bandwidths varying from around 20 to 50 nm full width half maximum. The stimulus was back-illuminated by various combinations of these five LEDs. The LEDs were controlled using an Arduino electronics platform and the time-averaged intensities were adjusted using pulse width modulation. In other words, the intensity of each of the LEDs was always on or off but the perception of different sources with differing intensities was created by varying the relative on and off times at a high speed, so no flicker was perceived.

The refractive state of the eye was measured using the PlusOptix PowerRef 3 autorefractor while subjects were viewing the accommodative stimulus. The operation of the device is based on tracking of Purkinje images. The validity of using such devices is discussed by (Gehring, Haensel, Curtiss, & Roberts, 2022; Ghahghaei, Reed, Candy, & Chandna, 2019; Blade & Candy, 2006). It is important to calibrate the device, especially for absolute measurements and for subjects from different ethnic groups. We are more concerned with relative measurements but the autorefractor was still calibrated for each subject individually, using the procedure described in the supplement to (Fernandez-Alonso et al., 2024) based on the procedure described by Sravani et al. (2015).

### Participants

Twelve participants were recruited in total for this study and the study described in Fernandez-Alonso et al. (2024). Six of these were involved in this study but one was excluded as they showed no change in accommodation for different colors of monochromatic light.

It could be argued that this participant should have been included, but in this study we are essentially asking the question, “given people generally accommodate differently to different wavelengths (as discussed in our first paper on this topic (Fernandez-Alonso et al., 2024) how do those people who do accommodate to mixed stimuli?”; therefore, they were excluded.

The five participants whose data was used were between the ages of 23 and 28 years (mean = 26.2). They all had a visual acuity that was better than 0.3 logarithm of the minimum angle of resolution in each eye at both near and far distances without the need for spectacles or lenses.

This experiment involved monocular viewing as we want to investigate purely the effect of the color of the stimuli without cues from vergence, but we note that binocular vision can affect accommodation.

All participants gave informed written consent prior to taking part. The experiment was approved by the Department of Physics Ethics Committee at Durham University and conformed to the tenets of the Helsinki Declaration.

### Procedure

The LEDs were used in pairs and the effective luminance of each was adjusted to give different spectral profiles. These were red and green, red and blue, red and violet, orange and blue, orange and violet, and green and violet. For each pair there were seven mixtures, each with different relative luminances of the two LEDs. The luminances of each of the individual LEDs was adjusted so as to change the target luminance of each color in steps of 1.25 cd/m^2^, whereas the overall luminance was kept constant at approximately 10 cd/m^2^. This gave a total of 42 mixtures and 47 test spectra including the five individual LEDs. The luminance of each LED was 10 cd/m^2^ when set to just using one LED, although we used a standard V-lambda curve rather than adjusting for each participant.

Before each trial the orange LED was presented for 2.5 seconds as a pretrial stimulus. This was followed by the trial stimulus, in which any 1 of the 47 test spectra was presented for 2.5 seconds. The accommodation is measured when the pretrial orange light is used and then the experiment measures how the accommodation changes when the spectral content of the illumination changes. Each session contained one trial for each of the 47 test spectra in a randomized order. There was no break between trials meaning that each session lasted for 3 minutes and 55 seconds. Each participant completed at least 12 sessions, giving 12 repetitions for each of the test spectra.

Participants viewed the target monocularly with their right eye (their left eye was covered). They were instructed to look at the stimulus and keep the cross clear using the same type of effort as when reading a book. (We note that experiments have been done whereby the instructions given to subjects can affect accommodation) (Bradley, Xu, Thibos, Marin, & Hernandez, 2014). There was a pause button that they could press if they needed a break within a session. They were told that they could blink whenever they needed to within the session but that if their eyes were watering or they needed to blink a lot they should use the pause button. Between sessions observers were given a break for as long as they needed.

### Data analysis

The data points where the pupil was not found were treated as blinks and excluded along with data corresponding to 80 ms before and 160 ms after the blink. Data points with a refraction measure that was clearly erroneous (<−20 D or >20 D) were also excluded. The first 1,000 ms of data within each trial and and the first 1,500 ms within each pretrial were excluded to allow the participant time to accommodate.

The static refraction values were calculated as the mean of the remaining data for each trial and pretrial. The trial values were then normalised by subtracting the preceding pretrial value from each. This was based on the assumption that observers always accommodated to the same distance for the pretrial reference and this helped to correct for longer term measurement errors such as shifts in head position.

### Optical simulations

All of the simulations were run in Python or IDL using the wave approximation to model the eye.

### Monochromatic PSF

A complex amplitude of light was modeled in the pupil plane taking a top hat function to describe the amplitude and summation of Zernike terms to describe the phase (Goodman, 1968). The square modulus of the Fourier transform of the complex amplitude was calculated to give the point spread function (PSF), *P*(*x*, *y*). This was then convolved with the stimulus *S*(*x*, *y*) to give the final image *I*(*x*, *y*). The amplitude, *A*, is given by

(1) $$\begin{array}{c} \;A\left(k_{x},k_{y}\right)=\int_{0}^{R}rdr\int_{0}^{2\pi }d\theta \;e^{ik_{x}r\cos\left(\theta \right)+ik_{y}r\sin\left(\theta \right)+i\phi \left(r,\theta ;\Delta F\right)}\;\;\;\;\; \end{array}$$

where *k* is the wavenumber, *R* is the radius of the pupil, and ϕ(*r*, θ; Δ*F*) describes the phase of the wavefront across the pupil with coordinates *r* and θ for a defocus of Δ*F*. ϕ = 0 for a diffraction-limited eye, while ϕ(*r*, θ; Δ*F*) = π*r*^2^/(λΔ*F*) for an eye with defocus but no higher order aberrations. The PSF is proportional to

(2) $$P\left(x,y\right)=|A\left(k_{x},k_{y}\right){|}^{2}$$

while the retinal image is

(3) $$\begin{array}{c} \;I\left(x,y\right)=\int_{-\infty }^{\infty }dx^{'}\int_{-\infty }^{\infty }dy^{'}\;S\left(x^{'},y^{'}\right)P\left(x-x^{'},y-y^{'}\right)\;\;\;\;\; \end{array}$$

### Polychromatic PSF

Owing to LCA, the effective defocus Δ*F* varies with wavelength. To take this into account, we computed the polychromatic PSFs by generating the monochromatic PSFs at a series of wavelengths (400–700 nm in 5-nm steps). The LCA for each wavelength was calculated using the empirical equation (Thibos, Ye, Zhang, & Bradley, 1992),

(4) $$D\left(\lambda \right)=q_{1}-\frac{q_{2}}{\lambda -q_{3}},$$

where *D*(λ) is the additional defocus due to LCA, in diopters relative to 580 nm, λ is the wavelength in microns, *q*_1_ = 1.7312, *q*_2_ = 0.63346 and *q*_3_ = 0.21410. *D*(λ) was added on to the baseline defocus value to obtain the total defocus Δ*F*, and this was used to compute the monochromatic PSF for wavelength λ according to Equation 2. Each monochromatic PSF was then weighted by the relevant test spectrum and the luminous efficiency function, and summed across all wavelengths to give the polychromatic PSF for the particular baseline defocus under consideration. This methodology works for sources that are spectrally homogeneous (Ravikumar, Thibos, & Bradley, 2008).

### Visual Strehl ratio

The visual Strehl ratio (VSR) is based on Strehl ratio, which is the peak intensity of the PSF of the optical system divided by the peak intensity of a diffraction-limited PSF for the same pupil size. Given that the peak intensity of the PSF is effectively the area under the modulation transfer function (MTF) then an alternative equation for the Strehl ratio is given by

(5) $${\text{SR}}_{\text{MTF}}=\frac{\int_{-\infty }^{\infty }\text{MTF}\left(f_{i}\right)df_{i}}{\int_{-\infty }^{\infty }{\text{MTF}}_{\text{DL}}\left(f_{i}\right)df_{i}}\text{,}$$

where MTF(*f*_*i*_) is the Fourier amplitude spectrum of the polychromatic PSF, MTF_DL_(*f*_*i*_) is the MTF for the equivalent diffraction-limited eye, and *f*_*i*_ is the spatial frequency in the image plane. The VSR is similar except the MTF is weighted by the spatial frequencies that can be processed by the visual system given by the neural contrast sensitivity Function (nCSF). This can be expressed as

(6) $$\text{VSR}=\frac{\int_{-\infty }^{\infty }\text{nCSF}\left(f_{i}\right)\text{MTF}\left(f_{i}\right)df_{i}}{\int_{-\infty }^{\infty }\text{nCSF}\left(f_{i}\right){\text{MTF}}_{\text{DL}}\left(f_{i}\right)df_{i}}\text{,}$$

where the nCSF was taken from Mannos and Sakrison (1974) and given by

(7) $$\text{nCSF}\left(f_{i}\right)=\frac{\text{CSF}(f_{i})}{\text{MTF}(f_{i})}\text{,}$$

where the CSF

(8) $$\;\text{CSF}\left(f_{i}\right)=2.6\left(0.0192+0.114sf_{i}\right)e^{-{\left(0.114sf_{i}\right)}^{1.1}}\text{.}$$

The advantage of the VSR over the Strehl ratio is that, as well as taking into account the effect of the optics of the eye on the resultant image quality, it aims to capture the effect of the subsequent neural processing that occurs.

### Encircled energy metrics

Encircled energy is defined as the percentage of the total energy falling within a circle that is centred at the peak of the PSF. One measure of encircled energy is known as light-in-the-bucket (LIB) (Thibos, Hong, Bradley, & Applegate, 2004). Here, the region of interest is the area taken up by the core of the diffraction limited PSF. This can be defined as

(9) $$\text{LIB}=\int_{0}^{2\pi }\int_{0}^{\text{DL}\;\text{core}}\text{PSF}\left(r_{i},\theta_{i}\right)dr_{i}d\theta_{i}\text{,}$$

where the domain of integration is the core of the diffraction limited PSF for the same pupil diameter and PSF is the normalised PSF (sum of energy = 1).

Another such metric is R50. Here, the value of interest is the radius of the circle containing 50% of the energy in the PSF. A smaller value of R50 indicates a more compact PSF and, therefore, a better image quality. Thibos et al. (2004) defined R50 as being equal to the radius, *r*, when

(10) $$\int_{0}^{2\pi }\int_{0}^{r}\text{PSF}\left(r_{i},\theta_{i}\right)dr_{i}d\theta_{i}=0.5\text{.}$$

## Results

### Experimental results

**Figure 4.**
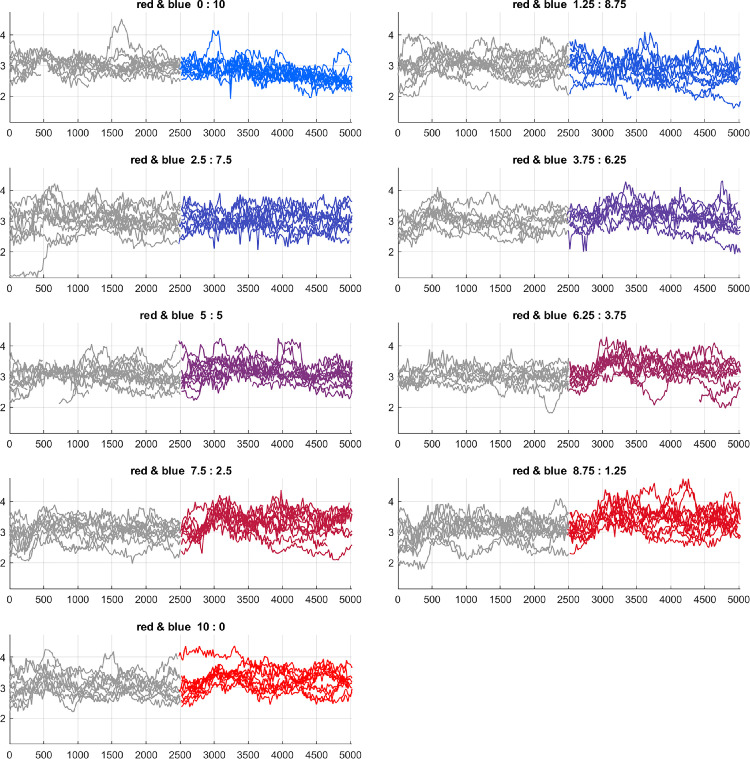
Example raw data for a single subject. The *x* axis shows the number of data points (and hence is a measure of time) and the *y* axis shows the measured accommodation in dioptres. There are a number of lines for each graph corresponding to different trials. Each plot is for a different mix of red and blue light as shown in the title of each panel (units are cd/m^2^). The left hand side of the graphs (colored in grey) shows the data when the intial orange light was displayed and the right hand side shows the data when the light switched to the mixed color.

Figure 4 shows an example set of raw data showing the accommodation measured by the autorefractometer as a function of time for a range of mixtures of blue and red light. The left hand sides of the traces (shown in grey) are the initial state with orange light and the right hand side—which are colored—show the final states. It can be seen that the data is both noisy and the changes in accommodation we are measuring are small but sufficient for data analysis.

**Figure 5.**
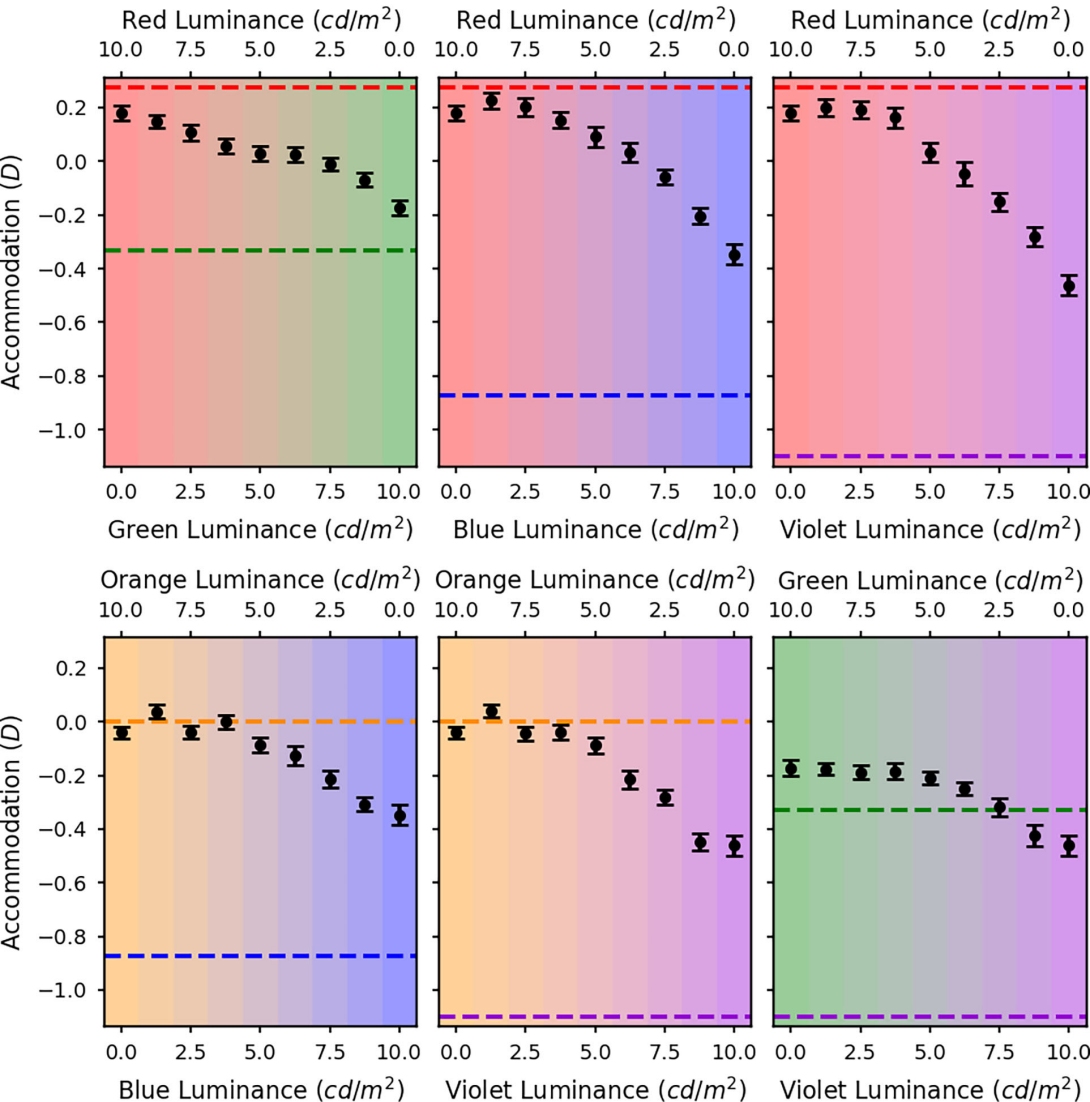
Accommodation versus luminance of one of the colors in a two-color display where the total luminance was constant. The black circles show the relative mean static accommodation responses of all five subjects plotted against the luminances of the two LED sources. The different graphs are for the different LED pairs: red and green (top left), red and blue (top middle), red and violet (top right), orange and blue (bottom left), orange and violet (bottom middle), and green and violet (bottom right). The error bars represent the standard error of the mean. The dashed lines indicate the accommodation needed to bring the defocus for the peak wavelength of each LED to zero.

Figure 5 shows the average static accommodation measures across all of the participants for each of the spectra. Each panel represents one mixture of two wavelengths. The dashed lines show the accommodative demands for those two wavelengths, relative to demand for the orange LED; we will refer to these as the primary demands. The leftmost and rightmost data points in each panel represent the average accommodative response to the two individual LEDs when each is presented in isolation; we will refer to these as the primary responses. Taking the top left panel in Figure 5 as an example (red and green mix) the horizontal green and red dashed lines are where, we would expect the eye to focus at each extreme of 100% red (left of figure) and 100% green (right of figure), as described by Equation 4. Compared with orange light the eye must increase accommodation by around 0.2 D for red light and decrease by about 0.35 D for green light. For a mixture of red and green, it is somewhere in the middle. The results show relative accommodation where 0 D is defined to be for orange wavelengths.

In each case, the difference in primary responses is in the expected direction, though by less than needed to compensate fully for LCA. Analyses of variance were performed for each pair of LEDs for each participant. In all cases these confirmed a significant main effect of luminance ratio. In the Supplementary Material, we present similar results obtained in a different experiment, using an OLED display and a visual task.

**Figure 6.**
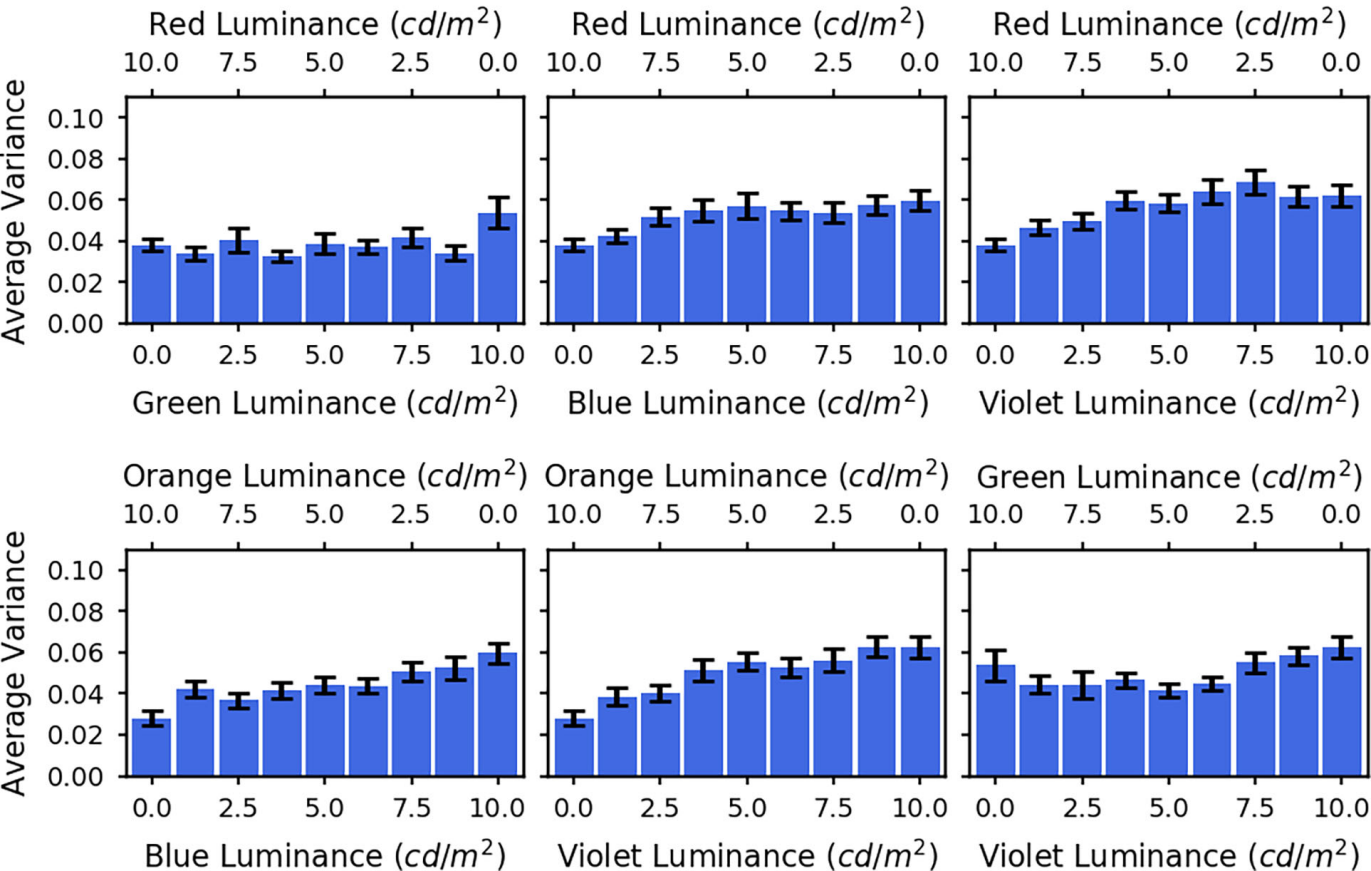
The bars show the within trial variances averaged across all five observers for each of the spectra. The different graphs are for the different LED pairs: red and green (top left), red and blue (top middle), red and violet (top right), orange and blue (bottom left), orange and violet (bottom middle), and green and violet (bottom right). The error bars represent the standard error of the mean.

Because the datapoints in Figure 5 represent averages across trials and across participants, it is possible that individual participants were only focussed on one of the two primaries in the mixture and switched from one to the other at a certain luminance ratio. The apparently graded response could result from differences in where this step occurred on different trials and for different participants. It is also possible that even within a particular trial, the individual’s accommodation may switch between the two primary responses. To examine whether this was occurring, we calculated the within-trial and between-trial variance for each spectrum for each observer. The average variances for all observers are shown as a function of luminance ratio in Figures 6 (within trial) and 7 (between trial). If the observers’ accommodation responses were switching between the two primaries, then we would expect a symmetrical trend in the variance, with an increased variance toward the centre where the mixtures are most even, and miminal variance at the ends where accommodation should be fixed at one primary. There is no evidence of such a symmetrical trend in the variances. Between-trial variance is relatively independent of luminance ratio, while within-trial variance tends to increase with the luminance of the shorter wavelength, consistent with our previous findings that accommodation is more variable for shorter wavelengths (Fernandez-Alonso et al., 2024). We conclude that the accommodation results were not due to switching. Rather, for mixture spectra observers show a weighted average of the two primary response, with weights depending on the relative luminance of the two primaries.

**Figure 7.**
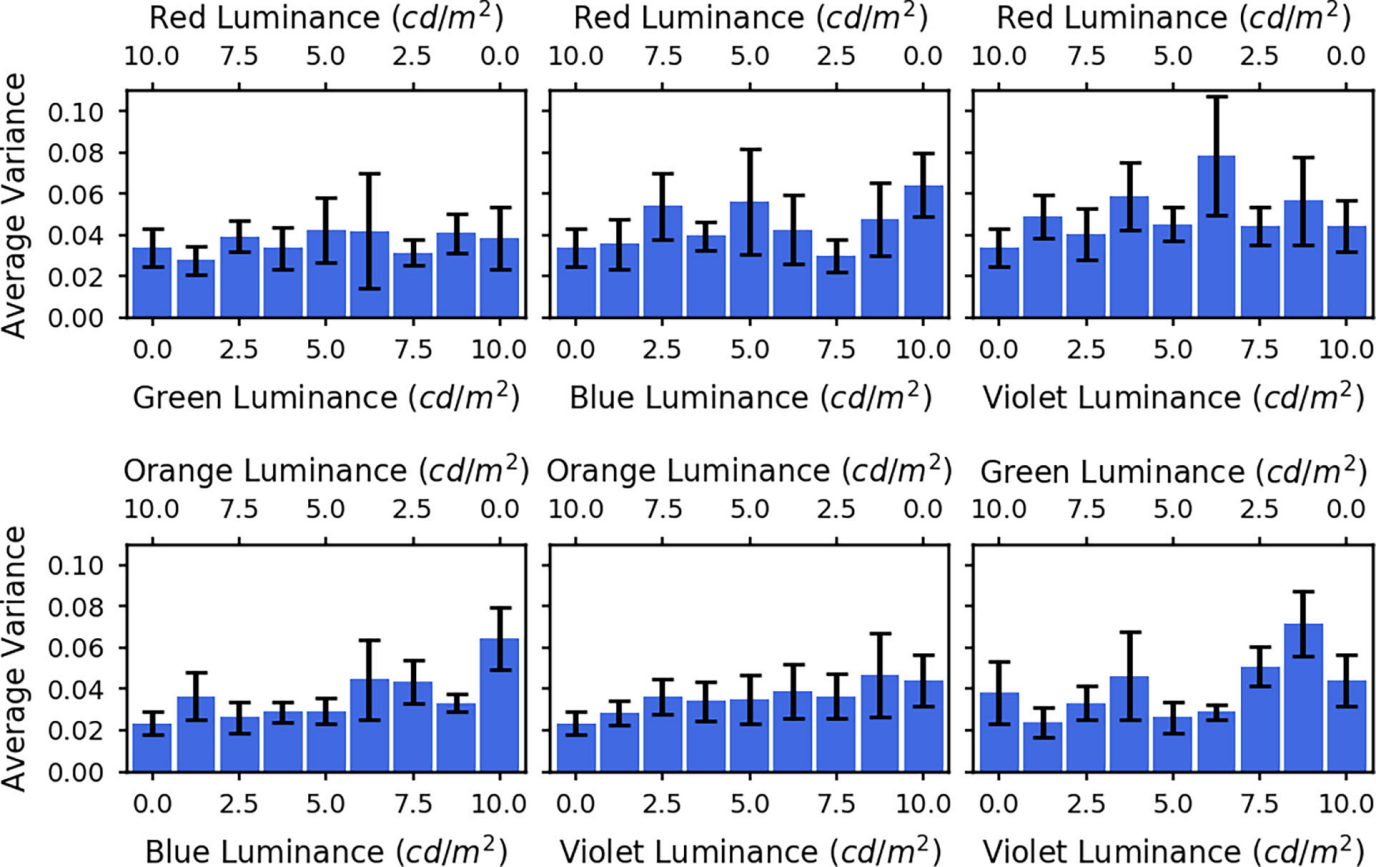
The bars show the between trial variances averaged across all five observers for each of the spectra. The different graphs are for the different LED pairs: red and green (top left), red and blue (top middle), red and violet (top right), orange and blue (bottom left), orange and violet (bottom middle), and green and violet (bottom right). The error bars represent the standard error of the mean.

Levene’s tests were also used to assess the difference in variances for each pair of LEDs, for each of the participants. Six of the 24 tests showed an overall significant difference between variances across the different luminance mixtures. A linear contrast was then performed on the *Z* values to compare the variances for the mixed spectra with those for the single LEDs. This was insignificant in all cases.

### Discussion of experimental results

As noted, the difference between the response to monochromatic light at each extreme (the primary response) is less than predicted by LCA, represented by the horizontal dashed lines in Figure 5 (the primary demand). Thus accommodation is not compensating fully for the differences in demand owing to LCA. Although artefactual leads and lags are well-known to occur in autorefractors (Labhishetty, Cholewiak, Roorda, & Banks, 2021), we do not think these are likely to be responsible here. We found previously (Fernandez-Alonso et al., 2024) that the extent to which accommodation compensates for LCA depends on viewing distance: at a distance of 0.5 D, we found no significant differences in the accommodation to different wavelengths, whereas at approximately 4.5 D, accommodation varied nearly to the full extent predicted by LCA. Our stimuli here were at 3 D, where our previous work found errors similar to those observed here, of around 0.5 D. The relatively small differences at the extremes using monochromatic light might also be due to the depth of field of the eye (around 0.25 D). Another possible reason is that the optimal accommodation response may not actually be the one that minimises the defocus. Certain monochromatic aberrations of the eye, such as spherical aberration, interact with defocus so that a certain magnitude of defocus actually helps to cancel out the blur in the retinal image caused by the other aberrations. Spherical aberration also varies with the accommodative state of the eye and therefore the optimal amount of defocus will vary depending on the accommodative state, which could explain the effect of distance we previously reported. Regardless of the reason, we now turn to the critical question of what occurs in between these two extremes.

The first hypothesis discussed in the Introduction was that accommodation would always be at one of the two primary responses for the LEDs making up the mixture (Charman, 1989) and this hypothesis clearly does not fit our data. This difference with Charman (1989) is perhaps unsurprising; in their stimuli, the red and the blue primaries were not mixed: there was either a red C on a blue background or vice versa. In our stimuli, in contrast, the two primaries were mixed with a diffuser, so accommodating to one of the individual components was not such an obvious response.

Our second hypothesis was that, as long as the difference in defocus between the two LEDs owing to LCA was less than 1.1 D, there would be a linear transition in the accommodation response through the mixtures. For the red and green, orange and blue, orange and violet, and green and violet mixtures, the separation between the peak wavelengths due to LCA is less than 1.1 D. Figure 5 shows that although there is a smooth transition in accommodation across the mixtures, in most cases, there is an asymmetry in the responses toward the longer of the two wavelengths meaning that the response is not quite linear. The smallest dioptric separation is between the red and green LEDs and in this case the transition in the accommodation responses across the mixtures does seem to be roughly linear. For this reason, the findings do at least somewhat agree with the predictions based on the MacKenzie and Watt (2010) and Altoaimi et al. (2018) studies.

### Simulation results

**Figure 8.**
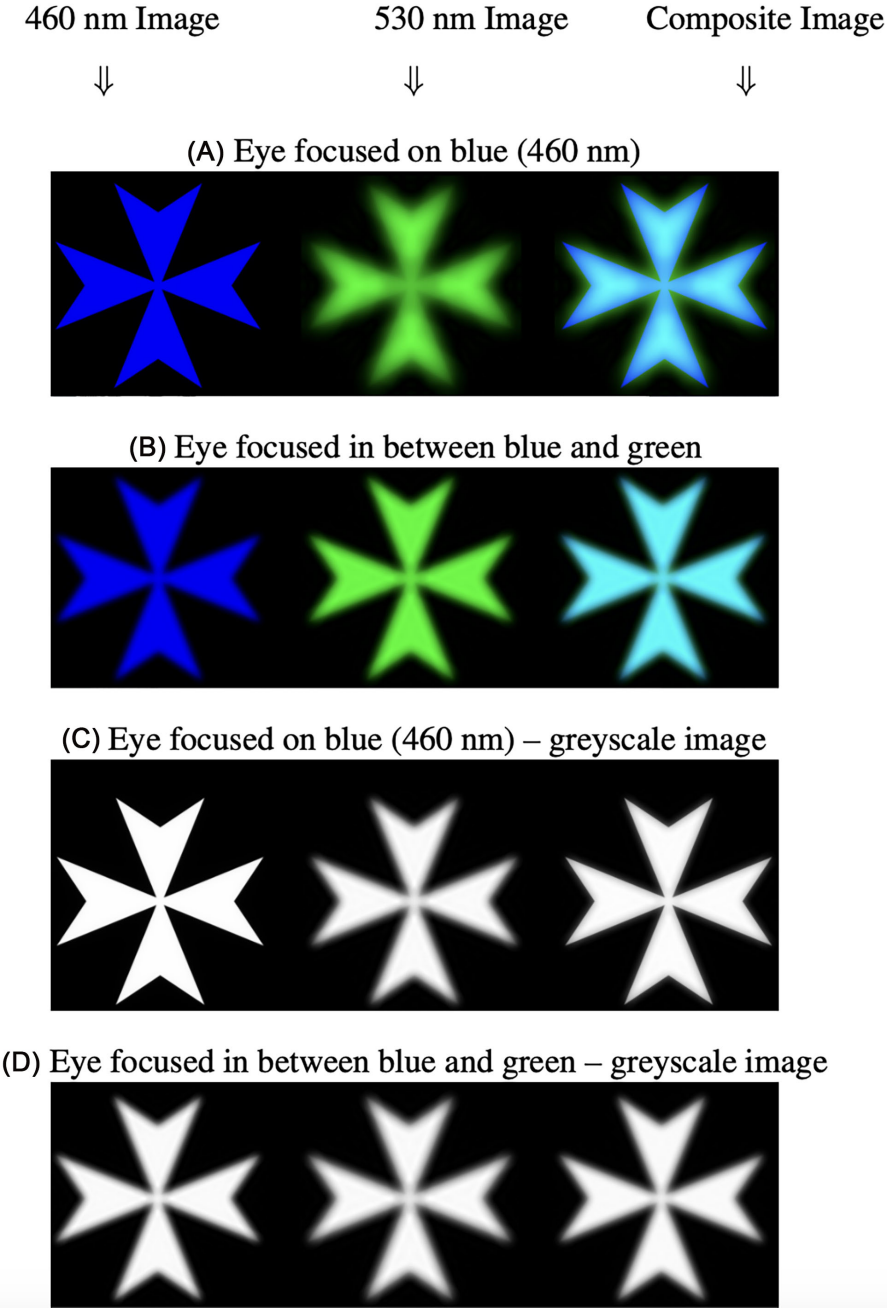
Simple simulation showing images on the retina of a cross pattern illuminated by two monochromatic wavelengths (460 nm and 530 nm). (A and B) Color images where the 460 nm is plotted using the blue channel and the 530 nm by the green channel. (C and D) The same results plotted using a grayscale. These results are a simplified guide to the problem. The left column shows the image of the 460 nm light, the middle column shows the 530 nm light, and the right column shows the sum of the two. (A and C) The eye is focused on one color (460 nm). Clearly that color is in focus and the other is blurred. (B and D) The eye focused in the middle and both colors appear slightly blurred. The interesting observation is that the sum of the two in the third column is apparently better when the eye is focused on one color rather than in the middle. All the images are plotted using a normalized greyscale. The field of view (of one of the crosses) is 1.6°. For realistic viewing the eye should be at a distance = 54 × the size of one of the crosses.

**Figure 9.**
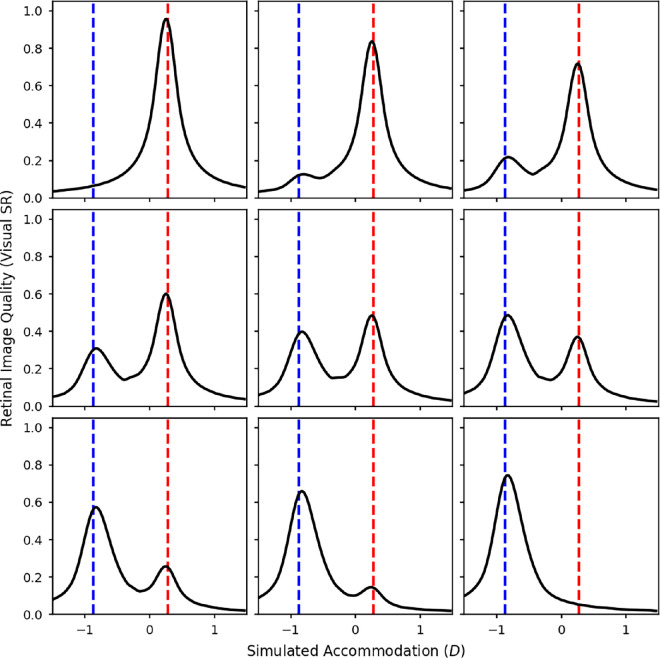
VSR calculated from a wave optics model of the eye with a 5-mm pupil over a range of defocus values, relative to 580 nm (the orange LED in our experiments). The test spectra are mixtures of the red and blue LEDs. The luminance ratio of these two sources was varied in nine equal steps from completely red (top left) to completely blue (bottom right). The red and blue dashed lines indicate the accommodative response needed to correct the LCA at the peak wavelengths of the red and blue LEDs.

On first consideration, focusing midway between two superimposed depth planes seems reasonable. However, this strategy does not maximise image sharpness. As a simple demonstration, Figure 8 shows the simulated light distribution on the retina for a Maltese cross stimulus made up of two monochromatic wavelengths; 460 nm and 530 nm. These correspond with the blue and green LEDs in the experiment. We choose these two colors as the LCA varies more in the blue and so the results are easier to see. The field of view of the target was 1.6°. The results are shown in Figure 8. Figure 8 (top) attempts to render the colors, while the lower panels are in grayscale to better appreciate the stimulus as perceived by achromatic mechanisms. Figures 8A and 8C depict the stimulus when the eye is focused on the blue image. The blue image therefore appears sharp while the green image is slightly blurred. However, the superposition of both images is barely degraded. Figures 9B and 8D depict the stimulus when the eye is focused in between the two wavelengths. Now, both images are slightly blurred and so the superposition is also degraded. This indicates that the image quality might be better when the eye is focused on one wavelength rather than in the center.

#### Maximizing overall image quality

To explore this more formally, we modeled the accommodation expected for each stimulus, assuming that the visual system aims to maximise retinal image quality. In the first instance we used VSR, Equation 6, as our measure of image quality, as this has been shown to be a very good predictor of visual acuity (Cheng, Bradley, & Thibos, 2004; Marsack, Thibos, & Applegate, 2004; Thibos et al., 2004).

Figure 9 shows the calculated VSR as a function of defocus for the test spectra of various mixtures of the red and blue LEDs for a pupil size of 5 mm, which was typical in our experiment. Defocus is defined relative to 580 nm, the orange LED, whereas the blue and red dashed lines mark where the blue and red LEDs respectively are in focus, that is, the primary demands. The top-left/bottom-right panels are for monochromatic red/blue light, respectively, the middle panel is for the mixture with equal luminance, and the remaining panels show mixtures with more or less red light. In each case, the VSR was calculated from polychromatic MTFs weighted by the given mixture spectrum and the luminous efficiency function, as described in the Methods (Equation 6).

It is clear from Figure 9 that there are two separate peaks in VSR, one around where the peak wavelength of the red LED is in focus and the other around where the peak wavelength of the blue LED is in focus. As the luminance of the blue LED increases the peak in the VSR at the blue wavelengths also increases, and vice versa. The accommodative response which maximises the VSR for a given spectrum is the defocus value resulting in the peak VSR. From Figure 9, whenever the red LED has higher luminance, this will be at the red demand, jumping to the blue demand whenever the blue LED has higher luminance. There is no situation where VSR is maximized by accommodating in between the two primary demands.

**Figure 10.**
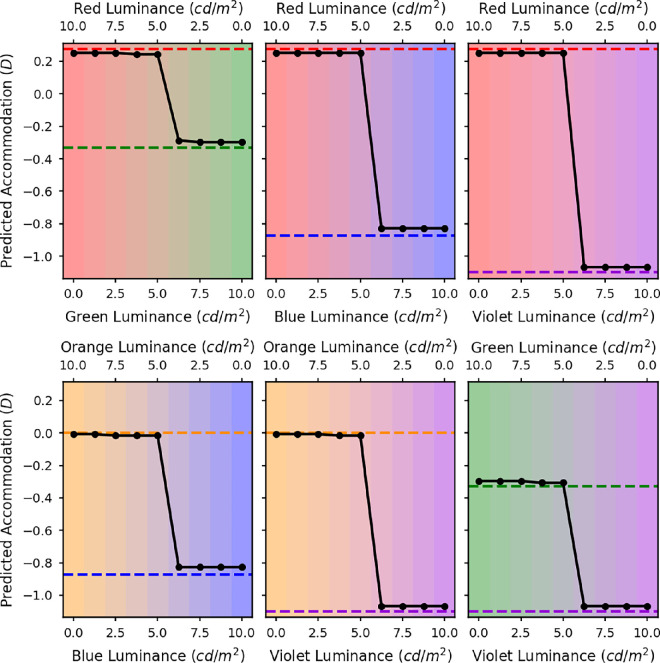
The predicted accommodation responses for maximizing VSR in the luminance channel for a 5-mm pupil. The *x* axis represents the luminances of the two LED sources. The different graphs are for the different LED pairs: red and green (top left), red and blue (top middle), red and violet (top right), orange and blue (bottom left), orange and violet (bottom middle), and green and violet (bottom right). The dashed lines indicate the accommodative response needed to correct the LCA at the peak wavelengths of the LEDs.

Figure 10 shows the accommodative response which maximises VSR for each of the LED mixtures. The *x* axes of these graphs corresponds with the relative luminances of the two LEDs. The *y* axes of these graphs are equivalent to the *x* axes from Figure 9, with the location of the symbols marking the defocus value corresponding to the peak VSR. The predicted focus values for all of these combinations show the same step pattern as the red and blue mixture, meaning that in all cases the VSR is optimised when one of the two LEDs is in focus.

In these simulations, we assumed that the accommodation system finds and accommodates to the overall peak in VSR, which is the highest of the two peaks typically shown in Figure 9. However, if the visual system was using a trial and error method, for example by using the microfluctuations in defocus to judge the direction of the accommodation response needed, it is conceivable that the actual accommodation response could get stuck in a local maximum rather than reliably finding the overall peak in image quality. Either way, we still predict that accommodation matches either the red or blue primary demand. This model never predicts accommodation midway between the two.

Clearly, this does not match the measured accommodation results in Figure 5, where for many luminance ratios participants accommodate in between the two primary demands. This suggests that observers are not actually maximizing image quality.

#### Different image quality metrics

It is possible that the simulation results described are just due to the specific image quality metric chosen. Perhaps if we used an image quality metric other than VSR we would see different results. In order to ensure that this was not the case we ran exactly the same simulation as that described but with different image quality metrics: LIB, Equation 9, and R50, Equation 10.

We ran the same simulations as described, but this time defining the predicted accommodation response as either the point at which the LIB value was the highest or the point at which the R50 value was lowest. The results were very similar to the VSR predictions. There was generally a reduced image quality in between the two LEDs, and the predicted accommodation was always around one of the individual LEDs and not in between the two.

These findings further support the idea that the way that observers were accommodating to these mixed chromatic stimuli was not maximizing the image quality.

#### Maximizing contrast at different spatial frequencies

**Figure 11.**
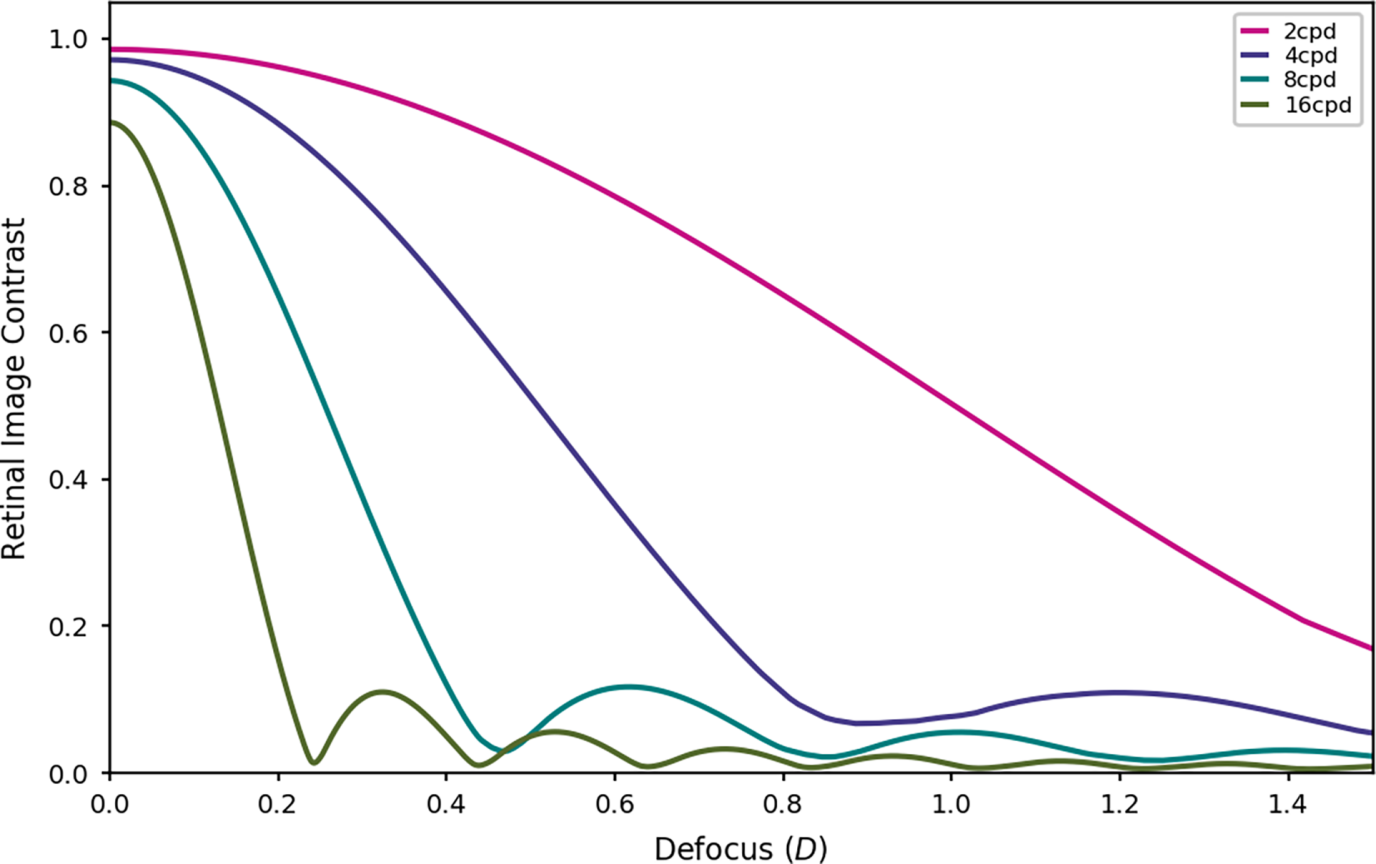
Simulated modulation transfer as a function of defocus plotted for 2 (magenta), 4 (purple), 8 (cyan), and 16 (green) cycles per degree (cpd).

Figure 11 shows modulation transfer as a function of defocus, for different spatial frequencies. Low frequencies could be used to drive accommodation as contrast is a monotonic function of defocus up until large values. High frequencies show a more rapid change in contrast—but then oscillate—but they could play a role in fine tuning accommodation. It is still unclear which spatial frequencies are most important for accommodation. However, a large amount of the research seems to indicate that lower spatial frequencies between around 3 to 6 cpd play the most crucial role.

The set of simulations described here were carried out in the same way as the ones described. However, once the polychromatic MTFs had been calculated, instead of calculating the VSRs from these, the contrasts were simply read off for a series of discrete spatial frequencies.

**Figure 12.**
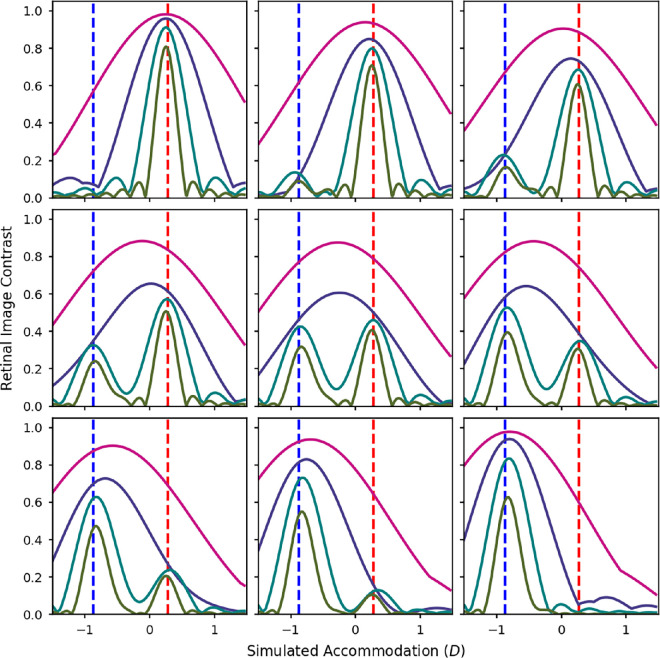
Contrast ratios calculated from a wave optics model of the eye with a 5mm pupil over a range of defocus values, at 2 (magenta), 4 (purple), 8 (cyan), and 16 (green) cpd. The contrast ratios were calculated from polychromatic MTFs weighted by each of the test spectra and the luminous efficiency function. The test spectra were mixtures of the red and blue LEDs. The luminance ratio of these two sources was varied in nine equal steps from completely red (top left) to completely blue (bottom right). The red and blue dashed lines indicate the accommodative response needed to correct for the LCA at the peak wavelengths of the red and blue LEDs.

**Figure 13.**
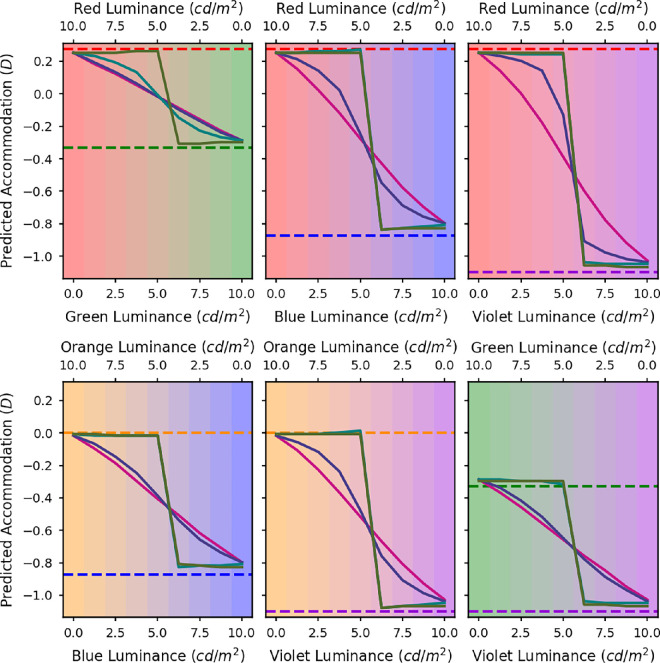
The predicted accommodation responses for maximising retinal image contrast in the luminance channel for spatial frequencies of 2 (magenta), 4 (purple), 8 (cyan), and 16 (green) cpd with a 5-mm pupil. The *x* axis represents the luminances of the two LED sources. The different graphs are for the different LED pairs: red and green (top left), red and blue (top middle), red and violet (top right), orange and blue (bottom left), orange and violet (bottom middle), and green and violet (bottom right). The dashed lines indicate the accommodative response needed to correct the LCA at the peak wavelengths of the LEDs.

Figure 12 shows the simulated retinal image contrasts at 2, 4, 8, and 16 cpd for mixtures of the red and blue LEDs. For each of the LEDs on their own (top right and bottom left), the optimum focus point was almost identical for all of the spatial frequencies and corresponded to the primary demand for that LED.

For the mixtures, the general pattern for the higher spatial frequencies of 8 and 16 cpd was similar to that found for the VSR. For these higher spatial frequencies, ith there were always two main peaks in contrast corresponding with the two peaks in the spectrum. As with the VSR the peak corresponding with the red LED increased as more red was added, and vice versa for the peak corresponding with the blue LED.

However, as the spatial frequency reduces, the lines become smoother and the peaks merge into one, so there are no longer two distinct peaks in contrast. For 2 cpd, there was just a single peak that moved more toward red wavelengths when more red light was added and toward blue wavelengths when more blue light was added. For the mixture with equal luminances of red and blue, the optimum focus position for maximizing contrast at 2 cpd is actually right in the middle of the two LEDs.

This is interesting because the point at which we might focus if we were maximizing contrast in the low spatial frequencies actually corresponds with a decrease in the contrast for the higher spatial frequencies. This means that, for these types of stimuli, accommodating to maximize contrast in lower spatial frequencies, a tactic that would normally maximize contrast across all spatial frequencies, actually results in a loss of contrast at higher spatial frequencies and, therefore, a degradation in the fine details of the image.

Figure 13 shows the predicted focus positions for maximising contrast at each of the four spatial frequencies. The separate graphs are for different LED combinations. Here we can see a clear step function for the higher spatial frequencies of 16 cpd and sometimes 8 cpd. As the spatial frequency decreases there is a slightly smoother sigmoid shape, and at 2 cpd the transition is even smoother and almost linear for some combinations. The only real difference between the different LED combinations is that the less dioptric separation there is between the two wavelengths due to the LCA of the eye, the smoother the curves tend to be, and the greater the dioptric separation, the more step-like the curves tend to be.

The measured accommodation responses in Figure 5 show a relatively smooth line across the mixtures. This seems to correspond best to the model predictions for lower spatial frequencies of 2 or 4 cpd in Figure 13. It implies that, for the mixture spectra where participants accommodated in between the two primary responses, they would have experienced very low contrast for the higher spatial frequency components of the image. Looking at the middle panel of Figure 12 we can see that if we focus at the peak in contrast for 2 cpd this would result in a dramatic reduction in contrast at 16 cpd. However, if we were to focus at the peak contrast for 16 cpd, the reduction in contrast at 2 cpd would be much less dramatic.

As mentioned elsewhere in this article, it may be that when there is a disagreement between the directions suggested by the high and the low spatial frequencies, the lower spatial frequencies tend to be favoured as they are generally more reliable (see Figure 11). Most of the mixed spectra used in this experiment did lead to disagreements in the optimum focus point for different spatial frequencies. This could explain why the behavior corresponds more closely with the predictions from lower spatial frequencies because these are generally more reliable and would usually maximize VSR in naturalistic broadband stimuli. It is only for our highly artificial spectra that the strategy misfires.

#### Using LCA as a cue

For the simulations described, we have made the assumption that the visual system is able to find the point at which the contrast or the overall image quality is at its highest. Another possibility is that the visual system might exploit LCA and compare the responses in different color channels when deciding where to accommodate.

Under natural circumstances, objects in the environment tend to be illuminated by relatively smooth, broadband spectra. In these cases, we can imagine that the optimal accommodation response would involve focusing in the wavelength range that we are most sensitive to (i.e., wavelengths around the peak of *V*(λ)). Because *V*(λ) is a combination of the spectral sensitivities of the L and M cones, this means that the ideal focus point would probably be somewhere in between the optimal focus for the L cone channel and the optimal focus for the M cone channel. Therefore, the point at which the image quality or contrast in the L and M cone channels is roughly balanced may provide a good approximation for the optimal focus position for natural illuminants. This will be referred to as the EquateLM rule. If the eye is focused in front of this, then the image quality or contrast would be better in the L cones than the M cones and, if the eye is focussed behind this, then the image quality or contrast would be better in the M cones than the L cones. A model along similar lines to this was proposed by Flitcroft (1990).

The simulations described in this section were run in a similar way to those for maximising overall image quality described above. However, instead of weighting the spectra by the luminous efficiency function, they were weighted by the L and M cone spectral sensitivities. Then, instead of finding the peak to predict accommodation, we found the point at which the image quality values for the L and M cones crossed over. In cases where there were multiple crossings, this was treated as ambiguous and there were multiple possible focus predictions.

**Figure 14.**
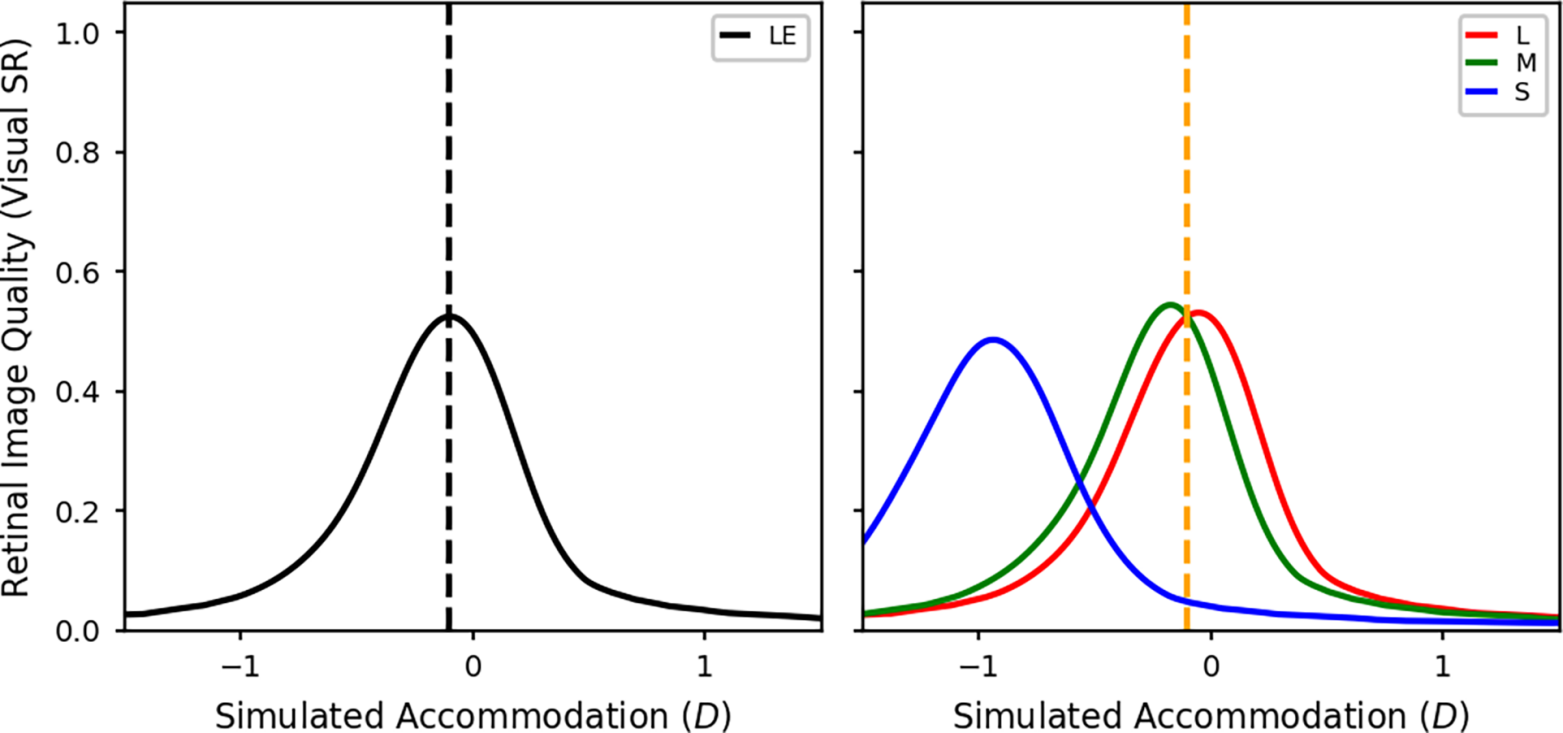
The VSRs for a range of defocus values with a D65 spectrum. The solid black line in the left graph shows the simulated VSR as a function of defocus related to the orange primary weighted by the luminous efficiency function. The black dotted line indicates the peak of this function and therefore the predicted accommodation response if the accommodation system were to maximize the VSR in the luminance pathway. The solid red, green and blue lines in the graph on the right show the simulated VSR weighted by the L (red), M (green), and S (blue) cone spectral sensitivities respectively. The dashed orange line indicates the defocus value at which the VSR is equal in the L and M cone channels and therefore the predicted accommodation response for a visual system equating image quality in the L and M cone channels. All of these simulations are for an eye with natural LCA and a 6-mm pupil. The optimum focus position is similar in the left and right panels.

**Figure 15.**
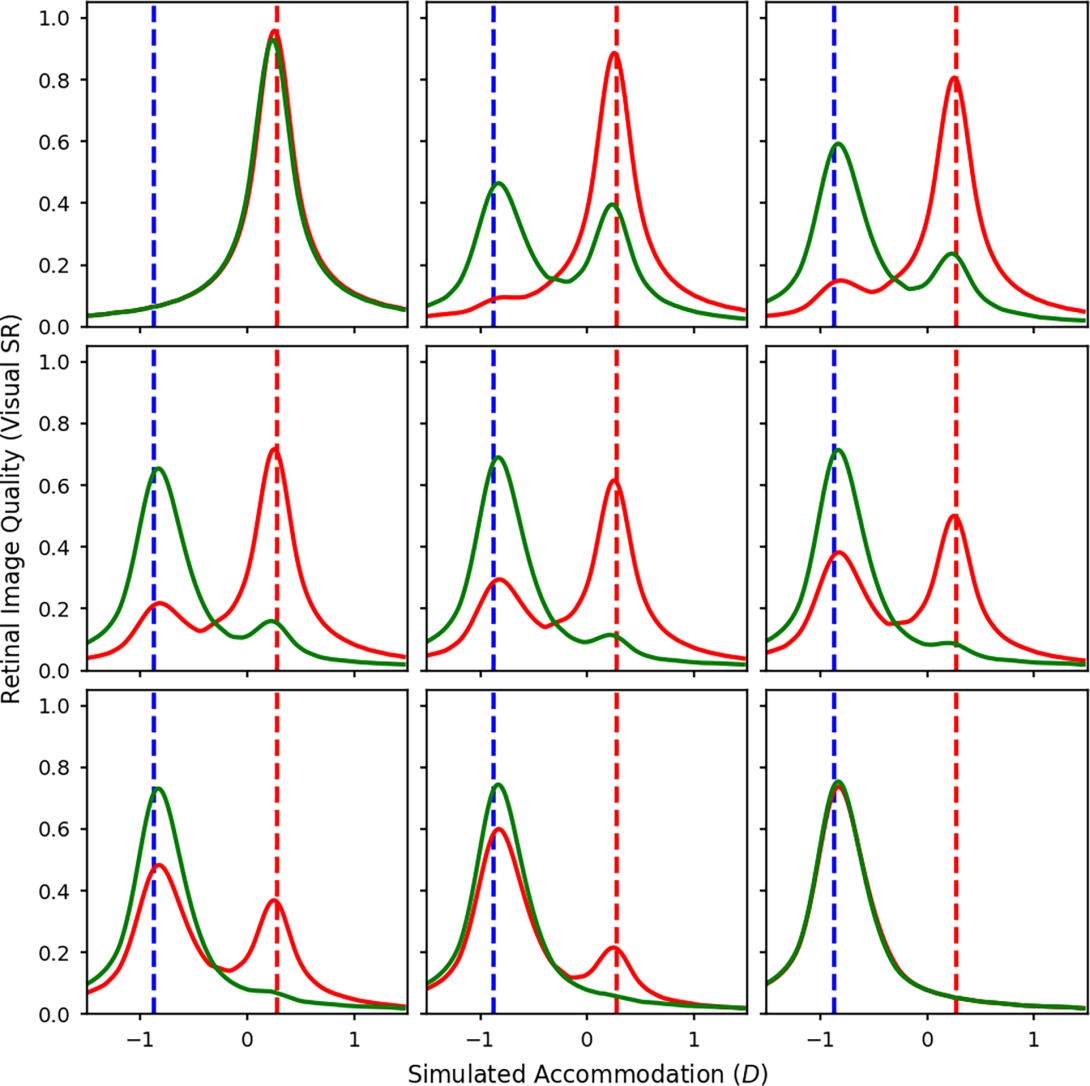
VSRs calculated from a wave optics model of the eye with a 5-mm pupil over a range of defocus values. The VSRs were calculated from polychromatic MTFs weighted by each of the test spectra and the L (red) and M (green) cone spectral sensitivities. The test spectra were made up of the spectra of the red and blue LEDs. The luminance ratio of these two sources was varied in nine equal steps from completely red (top left) to completely blue (bottom right). The red and blue dashed lines indicate the accommodative response needed to correct for the LCA at the peak wavelengths of the red and blue LEDs.

**Figure 16.**
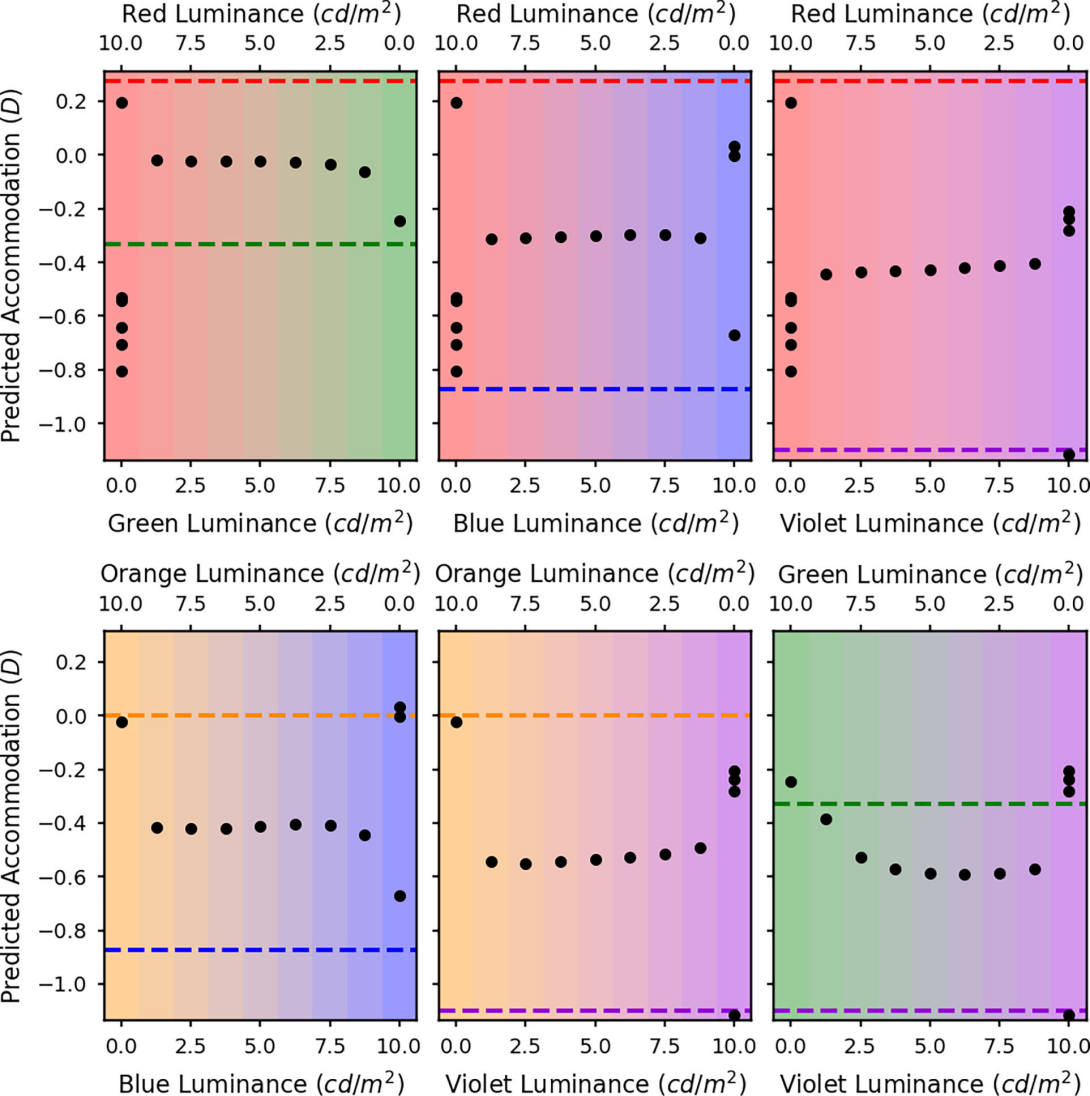
The predicted accommodation responses for VSR in the L and M channels with a 5-mm pupil. The *x* axes represent the luminances of the two LED sources. The different graphs are for the different LED pairs: red and green (top left), red and blue (top middle), red and violet (top right), orange and blue (bottom left), orange and violet (bottom middle), and green and violet (bottom right). The dashed lines indicate the accommodative response needed to correct the LCA at the peak wavelengths of the LEDs. At the extremes of the graph where there is only a single illumnant then the theory predicts multiple solutions, shown by the multiple points. Here LCA could not be a reliable cue.

Before running the simulations for our test spectra, we tested the assumption that the EquateLM rule would provide a good approximation for maximizing image quality in the luminance channel under natural broadband illuminants. Because the human visual system evolved under natural illuminants such as daylight, we would expect it to have selected a rule that would tend to maximize image quality under these spectra. To do this, we compared the simulated accommodation needed to equate L and M image quality with the simulated accommodation needed to maximize image quality in the luminance channel with the D65 illuminant spectrum (the CIE standard daylight).

The left panel of Figure 14 shows the VSR as a function of defocus for the D65 spectrum weighted by *V*(λ). The dashed vertical line shows the predicted accommodation if the VSR was optimised for the luminance channel, which is at −0.100 D. The right panel of Figure 14 shows the VSR as a function of defocus for the D65 spectrum weighted by the L, M, and S cone sensitivities. The orange dashed line shows the predicted accommodation for the EquateLM rule for VSR which is at −0.102 D. This confirms that for a natural broadband illuminant, the EquateLM rule gives similar results to optimizing the VSR and is thus a plausible candidate for human vision.

Next we investigated the predictions of this rule for the mixture spectra used in our experiments. Figure 15 shows the VSR in the L and M cone channels for each of the mixtures of the red and blue LEDs. For the red and blue LEDs on their own, shown in the top left and bottom right panels, respectively, it is clear that the image quality in the L and M channels is almost identical at all defocus values. This indicates that in cases where the illumination is monochromatic or very narrowband, then not surprisingly LCA cannot provide useful cues to accommodation. For the mixtures, there are two peaks in image quality for both the L and the M cone channels, one corresponding to the blue LED and the other to the red LED. The blue peak is more pronounced in the M cone channel as this is more sensitive to shorter wavelengths and the red peak is more pronounced in the L cone channel as this is more sensitive to longer wavelengths. For the mixtures, the image quality in the two channels always seems to cross almost directly in the middle of the two peaks.

The black filled circles in Figure 16 show the predicted accommodation responses for each of the experimental stimuli for the EquateLM rule with VSR. For some of the stimuli there were multiple crossings, which are shown as multiple predicted accommodation responses. For the individual LEDs there are often multiple crossings as the image quality is very similar for the two cone channels at all defocus values. Therefore, for the individual LEDs it is not clear where observers would accommodate if they were using the EquateLM rule. However, for the mixtures, the predicted accommodation response tends to be almost exactly in between the peak wavelengths for the two LEDs regardless of the relative luminances of the two LEDs. These results do not match the experimental results at all, suggesting that the human visual system is not using the EquateLM rule.

We also explored applying the EquateLM rule to individual spatial frequencies, in the same way as we did for VSR. For individual spatial frequencies, the predicted accommodation was still midway between the two primary demands, virtually independent of luminance ratio. Thus, this does not change the conclusion that the EquateLM rule cannot account for our experimental results.

#### Higher-order aberrations

All of the simulations described so far have been based on a model eye that is diffraction-limited (other than the LCA and induced defocus). However, real eyes also have higher-order monochromatic aberrations, which need to be considered. In general aberrations reduce the Strehl ratio—the peak intensity at best focus—but they also spread the light around leading to an increase in depth of focus. We performed some simulations exploring the effects of various realistic aberrations. We found that the major effect of aberrations was to smooth out the curves, making steps less abrupt. However, the basic pattern of results was the same: maximizing VSR predicts a much more abrupt transition between the two primary responses than observed in our data, while maximising contrast at low spatial frequencies gives a reasonably good account.

## Discussion

This paper aimed to understand where the visual system accommodates when the stimulus consists of two narrowband primaries presenting different accommodative demands due to LCA. This is a highly artificial situation, but arises in many modern visual displays.

Our key experimental findings, shown in Figure 5, are that as the luminance ratio of the primaries varies, accommodation varies smoothly between the two primary responses. As a secondary result, we also find that this variation is not quite linear if one of the primaries is blue or violet; rather, accommodation tends to stay at the response associated with the longer wavelength primary until the shorter wavelength primary has higher luminance.

The smooth variation between two superimposed accommodative demands, weighted by the relative luminance, is in line with previous reports using volumetric displays (MacKenzie & Watt, 2010) and multifocal contact lenses (Altoaimi et al., 2018), and at first sight seems a reasonable strategy. However, we have shown that it is hard to reconcile with many straightforward strategies for accommodation. The most obvious strategy is to optimise image quality, whether that is defined as Strehl ratio, the VSR, or another metric. However, as shown informally in Figure 8, such a strategy would predict accommodation on whichever wavelength has higher luminance. Accommodation would thus be a step function of luminance ratio, with a sharp switch as luminance ratio passes 1. If instead the visual system tried to balance the image quality in the L and M channels, accommodation would stay midway between the two primary responses, with very little dependence on luminance ratio. Both these are incompatible with the graded response actually observed.

The hypothesis which was most consistent with our results was that the visual system optimizes contrast ratio (or image quality) for low spatial frequency image components, in the range of 2 to 4 cpd.

There is debate in the literature as to which spatial frequencies are most important for the accommodative response. As we saw in Figure 11, lower spatial frequencies may be most informative initially when defocus is large, whereas the high spatial frequencies play an important role in fine tuning the response. In a theoretical study (Burge & Geisler, 2011) used a set of natural images together with a model of the human eye and early visual system to find a set of optimal filters for extracting defocus information from the environment. They found that these optimal filters were predominantly sensitive to relatively high spatial frequencies between 5 and 15 cpd. The idea that high spatial frequencies play an important role was supported by an experiment by Charman and Tucker (1978), who measured accommodation responses to sine wave gratings and found that in general responses were more accurate to gratings with higher spatial frequencies. However, there is also evidence to suggest that high spatial frequencies actually have little importance for accommodation. Studies have investigated accommodation responses to sinusoidal gratings with various spatial frequencies and found that optimal accommodation performance was achieved at low to intermediate spatial frequencies (between 3 and 5 or 1 and 7 cpd) (Owens, 1980; Stone, Mathews, & Kruger, 1993; Mathews & Kruger, 1994). Similarly Walsh and Charman (1988) investigated participants’ sensitivity to blur both for sinusoidal grating targets and for images with a broad spatial frequency bandwidth. They found that the blur sensitivity for the broadband images was most similar to that for gratings at around 5 cpd. Their results are consistent with ours in that subjects tended to focus between two planes, and the same is true for experiments with bifocal contact lenses (Altoaimi et al., 2018).

Of course accommodation is driven by many factors including vergence and our results are for monocular viewing. Further work is needed to look accommodation for mixed band stimuli under binocular conditions. We note that it has been reported that some subjects find it hard to accommodate at all for monocular viewing, which might explain the relatively large number of subjects in our study that were rejected (Lopez-Gil et al., 2013). It could be that the effects of color shown here are weaker with binocular vision, but more experiments are needed to test this.

Perhaps the most economical conclusion is that in normal conditions, accommodation uses a coarse-to-fine strategy, in which low spatial frequencies are used to remove large amounts of defocus and then high spatial frequencies are used to optimise image quality precisely. This strategy works well under natural illuminants, since image quality will peak at the same value of accommodation for all spatial frequencies. In our highly unnatural spectra, made up of mixtures of narrow-band primaries, this is not the case: high and low spatial frequency channels may not agree on the accommodation which maximises contrast, as shown in Figure 12. In this situation, our results and those of MacKenzie and Watt (2010) suggest that the low spatial frequency channels dominate, even though this strategy does not optimise overall image quality.

There is evidence that the eye does exploit the ability of LCA to provide a cue to the sign of accommodation. Kruger, Mathews, Aggarwala, and Sanchez (1993) measured accommodative responses to a sinusoidally moving target illuminated by either monochromatic or broadband white light, while the eye’s LCA was either normal, removed, or reversed. They found that gains were higher and phase-lags reduced when LCA was normal. We examined a strategy designed to exploit LCA by equating contrast in the L and M cone channels, but this could not account for our data. It may be that the signed LCA cue is useful in facilitating a rapid response to changes in accommodation, but that cues based on image quality are more relevant to the final value of static accommodation.

Our results have implications both for lighting design and for visual displays. If a surface is illuminated by two quasi monochromatic illuminants, for example, where a pinkish light is created by a combination of red and blue LEDs, or a display uses narrowband primaries, then the human visual system may be misled into inappropriate accommodation, which does not maximize the overall image quality. People will thus experience higher than usual amounts of blur. The actual effect in real lighting is likely to be small but this should nevertheless be borne in mind by lighting manufacturers and those generating content for visual displays.

## Conclusions

When viewing stimuli with spectra composed of two distinct peaks at distinct wavelengths, the static accommodation response tends to fall somewhere in the middle of that for each of the two peaks on their own. The response is biased toward the longer wavelength, but weighted by the relative intensities of the two peaks. This is predicted fairly well by a model that assumes the visual system tries to maximise image contrast at spatial frequencies of around 2 to 4 cpd, only maximizing contrast at higher spatial frequencies if this has no detrimental effect on the lower frequencies.

As a consequence, accommodation may not maximise overall image quality for such spectra, meaning that observers will perceive a slightly blurred image. This should be taken into account when designing and selecting lighting and display primaries.

## Supplementary Material

Supplement 1

## References

[bib1] Akeley, K., Watt, S. J., Girshick, A. R., & Banks, M. S. (2004). A stereo display prototype with multiple focal distances. In *ACM SIGGRAPH 2004 papers on - SIGGRAPH ’04* (Vol. 23, p. 804). New York: ACM Press. Available from http://portal.acm.org/citation.cfm?doid=1186562.1015804.

[bib2] Altoaimi, B., Almutairi, M. S., Kollbaum, P. S., & Bradley, A. (2018). Accommodative behavior of young eyes wearing multifocal contact lenses. *Optometry and vision science : official publication of the American Academy of Optometry, 95* (5), 416–427. 10.1097/OPX.0000000000001214 29683985

[bib4] Blade, P., & Candy, T. (2006). Validation of the powerrefractor for measuring human infant refraction. *Optometry and Vision Science,* 83(6), 346–53, doi:10.1097/01.opx.0000221402.35099.fb. 16772892 PMC2755530

[bib5] Bradley, A., Xu, R., Thibos, L., Marin, G., & Hernandez, M. (2014). Influence of spherical aberration, stimulus spatial frequency, and pupil apodisation on subjective refractions. *Ophthalmic and Physiological Optics,* 34(3), 309–320. 24397356 10.1111/opo.12114 PMC4114316

[bib6] Burge, J., & Geisler, W. S. (2011). Optimal defocus estimation in individual natural images. *Proceedings of the National Academy of Sciences of the United States of America,* 108(40), 16849–54, doi:10.1073/pnas.1108491108. 21930897 PMC3189032

[bib7] Charman, W. N. (1989). Accommodation performance for chromatic displays. *Ophthalmic and Physiological Optics,* 9(4), 459–463, doi:10.1111/j.1475-1313.1989.tb00959.x. 2631021

[bib8] Charman, W. N., & Tucker, J. (1978). Accommodation and color. *Journal of the Optical Society of America,* 68(4), 459–471, doi:10.1364/JOSA.68.000459. 671137

[bib9] Cheng, X., Bradley, A., & Thibos, L. N. (2004). Predicting subjective judgment of best focus with objective image quality metrics. *Journal of Vision,* 4(4), 310–321. Available from http://jov.arvojournals.org/article.aspx?articleid=2121812. 15134478 10.1167/4.4.7

[bib10] Coe, C., Bradley, A., & Thibos, L. (2014). Polychromatic refractive error from monochromatic wavefront aberrometry. *Optometry and Vision Science,* 91(10), 1167–1174, doi:10.1097/OPX.0000000000000361. 25105688

[bib11] DeHoog, E., & Schwiegerling, J. (2007). Position of white light best focus in the human eye. *Investigative Ophthalmology & Visual Science,* 48(13), 993. Available from http://iovs.arvojournals.org/article.aspx?articleid=2383876.

[bib12] Fernandez-Alonso, M., Finch, A. P., Love, G. D., & Read, J. C. A. (2024). Ocular accommodation and wavelength: The effect of longitudinal chromatic aberration on the stimulus–response curve. *Journal of Vision,* 24(2), 11, doi:10.1167/jov.24.2.11. PMC10910436 38411958

[bib13] Flitcroft, D. (1990). A neural and computational model for the chromatic control of accommodation. *Visual Neuroscience*. Available from https://www.researchgate.net/profile/Ian_Flitcroft/publication/21069500_A_neural_and_computational_model_for_the_chromatic_control_of_accommodation/links/57cd3bb208ae3ac722b53f8a.pdf. 10.1017/s0952523800000705 2085470

[bib14] Gehring, A., Haensel, J., Curtiss, M., & Roberts, T. (2022). Validation of the powerref 3 for measuring accommodation: Comparison with the grand Seiko WAM-5500A autorefractor. *Translational Vision Science & Technology,* 11(10), 25. 10.1167/tvst.11.10.25 PMC9587467 36255360

[bib15] Ghahghaei, S., Reed, O., Candy, T., & Chandna, A. (2019). Calibration of the plusoptix powerref 3 with change in viewing distance, adult age and refractive error. *Ophthalmic & Physiological Optics,* 4(39), 253–259, doi:10.1111/opo.12631. PMC6852400 31236979

[bib16] Goodman, J. (1968). *Introduction to fourier optics*. New York: McGraw-Hill.

[bib17] Ivanoff, A. (1949). Focusing wave-length for white light. *Journal of the Optical Society of America,* 39(8), 718.

[bib18] Kruger, P. B., Mathews, S., Aggarwala, K. R., & Sanchez, N. (1993). Chromatic aberration and ocular focus: Fincham revisited. *Vision Research,* 33(10), 1397–1411, doi:10.1016/0042-6989(93)90046-Y. 8333161

[bib19] Labhishetty, V., Cholewiak, S. A., Roorda, A., & Banks, M. S. (2021). Lags and leads of accommodation in humans: Fact or fiction? *Journal of Vision,* 21(3), 21–21, doi:10.1167/jov.21.3.21. PMC7995353 33764384

[bib20] Lopez-Gil, N., Martin, J., Liu, T., Bradley, A., Diaz-Munoz, D., & Thibos, L. N. (2013). Retinal image quality during accommodation. *Ophthalmic & Physiological Optics,* 33(4), 497–507. 23786386 10.1111/opo.12075 PMC3700370

[bib21] Lovasik, J. V., & Kergoat, H. (1988). Accommodative performance for chromatic displays. *Ophthalmic and Physiological Optics,* 8(4), 443–449, doi:10.1111/j.1475-1313.1988.tb01183.x. 3253638

[bib22] MacKenzie, K. J., & Watt, S. J. (2010). Eliminating accommodation-convergence conflicts in stereoscopic displays: Can multiplefocal-plane displays elicit continuous and consistent vergence and accommodation responses? In A. J. Woods, N. S. Holliman, & N. A. Dodgson (Eds.), *Is&t/spie electronic imaging* (pp. 752417–752417–10). International Society for Optics and Photonics. Available from http://proceedings.spiedigitallibrary.org/proceeding.aspx?articleid=776088.

[bib23] Mannos, J., & Sakrison, D. (1974). The effects of a visual fidelity criterion of the encoding of images. *IEEE Transactions on Information Theory,* 20(4), 525–536. Available from http://ieeexplore.ieee.org/xpls/abs_all.jsp?arnumber=1055250.

[bib24] Marsack, J. D., Thibos, L. N., & Applegate, R. A. (2004). Metrics of optical quality derived from wave aberrations predict visual performance. *Journal of Vision,* 4(4), 322–328, doi:10.1167/4.4.8. 15134479

[bib25] Mathews, S., & Kruger, P. B. (1994). Spatiotemporal transfer function of human accommodation. *Vision Research,* 34(15), 1965–1980, doi:10.1016/0042-6989(94)90026-4. 7941397

[bib26] Owens, D. (1980). A comparison of accommodative responsiveness and contrast sensitivity for sinusoidal gratings. *Vision Research,* 20(2), 159–167, doi:10.1016/0042-6989(80)90158-3. 7434577

[bib27] Ravikumar, S., Thibos, L. N., & Bradley, A. (2008). Calculation of retinal image quality for polychromatic light. *Journal of the Optical Society of America,* 25, 2395–2407. 18830317 10.1364/josaa.25.002395

[bib28] Sravani, N. G., Nilagiri, V. K., Bharadwaj, D. A., Shrikant R., Grosvenor, T., Choi, M., & Dahlmann-Noor, A. H. (2015). Photorefraction estimates of refractive power varies with the ethnic origin of human eyes. *Scientific Reports,* 5, 7976, doi:10.1038/srep07976. 25613165 PMC4303874

[bib29] Stone, D., Mathews, S., & Kruger, P. B. (1993). Accommodation and chromatic aberration: effect of spatial frequency. *Ophthalmic and Physiological Optics,* 13(3), 244–252, doi:10.1111/j.1475-1313.1993.tb00466.x. 8265165

[bib30] Switkes, E., Bradley, A., & Schor, C. (1990). Readily visible changes in color contrast are insufficient to stimulate accommodation. *Vision Research,* 30(9), 1367–1376, doi:10.1016/0042-6989(90)90010-i. 2219752

[bib31] Thibos, L. N., Hong, X., Bradley, A., & Applegate, R. A. (2004). Accuracy and precision of objective refraction from wavefront aberrations. *Journal of Vision,* 4(4), 329–351, doi:10.1167/4.4.9. 15134480

[bib32] Thibos, L. N., Ye, M., Zhang, X., & Bradley, A. (1992). The chromatic eye: A new reduced-eye model of ocular chromatic aberration in humans. *Applied Optics,* 31(19), 3594–3600, doi:10.1364/AO.31.003594. 20725330

[bib3] von Bahr, G. (1946). The visual acuity in monochromatic lights and in mixtures of two monochromatic lights. *Acta Ophthalmologica,* 24(2), 129–146.

[bib33] Walsh, G., & Charman, W. (1988). Visual sensitivity to temporal change in focus and its relevance to the accommodation response. *Vision Research,* 28(11), 1207–1221. 3253992 10.1016/0042-6989(88)90037-5

[bib34] Watt, S. J., Akeley, K., Girshick, A. R., & Banks, M. S. (2005). Achieving near-correct focus cues in a 3-D display using multiple image planes. In B. E. Rogowitz, T. N. Pappas, & S. J. Daly (Eds.), *Electronic imaging 2005* (pp. 393–401). International Society for Optics and Photonics. Available from http://proceedings.spiedigitallibrary.org/proceeding.aspx?articleid=857734.

[bib35] Wolfe, J., & Owens, D. A. (1981). Is accommodation colorblind? focusing chromatic contours. *Perception,* 10(1), 53–62, doi:10.1068/p100053. 7255083

