# Supplementary material for: Focusing on mixed narrow band stimuli: Implications for mechanisms of accommodation and displays
Source: J Vis. 2024 Sep 20;24(9):14. doi: 10.1167/jov.24.9.14 (PMC11421670; doi:10.1167/jov.24.9.14)
Supplement: Supplement 1 [file jovi-24-9-14_s001.pdf]

Supplementary Material

Our results in the main section suggest that accommodation is chosen so as to maximise contrast at lower spatial frequencies. This conclusion was based on free viewing. We speculated as to what would happen if observers were performing a task which required them to use high spatial frequencies. Would the strategy shift so as to maximise contrast at high spatial frequencies? If so, then we would expect to see accommodation switch abruptly from one primary response to the other, rather than the graded response shown in Figure 5.

To test this we used the OLED display described in (Fernandez-Alonso et al.,2024) placed at approximately 4.4 D to present square-wave gratings of 2.2, 9.1 and 18.1cpd, oriented at $\pm 45\,\mathrm{deg}$ to the vertical, as well as a radial ”starburst” pattern composed of multiple spatial frequencies. As before, the stimuli were presented in mixtures of two of the three OLED primaries. Participants were asked to view the stimuli and report the orientation of the grating, or report seeing the radial pattern. Performance was reliably over 90%.

Figure S1 shows the average static accommodation, averaged over participants, for the three pairs of primaries: red/blue, red/green, green/blue. Results are qualitatively similar to those of Figure 5. For the red/blue pair, where the primary demands are most different, the primary responses more closely track primary demand for the finer stripe patterns. This suggests that participants made more accommodative effort when the visual task was harder. However importantly, there is still no suggestion of a step-change in accommodation. Accommodation still changes in a graded way as a function of luminance ratio. Ironically this means that despite trying harder, participants were still not succeeding in maximising image quality. This confirms that the results in the main paper are not specific to free viewing, but also hold while participants are performing a visual task.

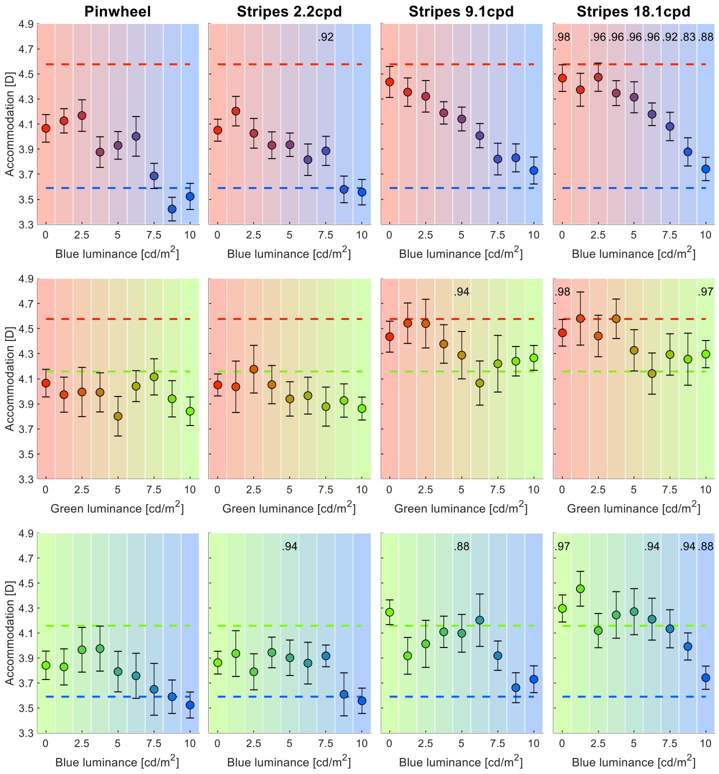

Figure S1: Experimental results showing accommodation for three of the color pairs (shown by the color in the panels for four different targets (shown by the four columns).
